# Structural insights into RNase J that plays an essential role in Mycobacterium tuberculosis RNA metabolism

**DOI:** 10.1038/s41467-023-38045-z

**Published:** 2023-04-20

**Authors:** Luyao Bao, Juan Hu, Bowen Zhan, Mingzhe Chi, Zhengyang Li, Sen Wang, Chan Shan, Zhaozhao Zhao, Yanchao Guo, Xiaoming Ding, Chaoneng Ji, Shengce Tao, Ting Ni, Xuelian Zhang, Guoping Zhao, Jixi Li

**Affiliations:** 1 State Key Laboratory of Genetic Engineering, School of Life Sciences and Huashan Hospital, Shanghai Engineering Research Center of Industrial Microorganisms, Engineering Research Center of Gene Technology of MOE, Fudan University, 200438 Shanghai, China; 2 State Key Laboratory of Genetic Engineering, School of Life Sciences, Fudan University, 200438 Shanghai, China; 3 Key Laboratory of Systems Biomedicine (Ministry of Education), Shanghai Center for Systems Biomedicine, Shanghai Jiao Tong University, 200240 Shanghai, China; 4 Key Laboratory of Synthetic Biology, CAS Center for Excellence in Molecular Plant Sciences, Shanghai Institute of Plant Physiology and Ecology, Chinese Academy of Sciences, 200032 Shanghai, China; 5 Shanghai Key Laboratory of Infectious Diseases and Biosafety Emergency Response, National Medical Center for Infectious Diseases, Huashan Hospital, Fudan University, 200040 Shanghai, China

**Keywords:** X-ray crystallography, Bacterial structural biology, Tuberculosis

## Abstract

Ribonucleases (RNases) are responsible for RNA metabolism. RNase J, the core enzyme of the RNA degradosome, plays an essential role in global mRNA decay. Emerging evidence showed that the RNase J of *Mycobacterium tuberculosis* (Mtb-RNase J) could be an excellent target for treating Mtb infection. Here, crystal structures of Mtb-RNase J in apo-state and complex with the single-strand RNA reveal the conformational change upon RNA binding and hydrolysis. Mtb-RNase J forms an active homodimer through the interactions between the β-CASP and the β-lactamase domain. Knockout of RNase J slows the growth rate and changes the colony morphologies and cell length in *Mycobacterium smegmatis*, which is restored by RNase J complementation. Finally, RNA-seq analysis shows that the knockout strain significantly changes the expression levels of 49 genes in metabolic pathways. Thus, our current study explores the structural basis of Mtb-RNase J and might provide a promising candidate in pharmacological treatment for tuberculosis.

## Abstract (web summary)

Here the authors report the crystal structures of RNase J from *Mycobacterium tuberculosis* in apo-state and complex with single strand RNA, revealing its physiological function in RNA metabolism.

## Introduction

Tuberculosis (TB) caused by *Mycobacterium tuberculosis* (Mtb) is the top-deadliest infectious disease worldwide and has posed a severe threat to human health for thousands of years^1,2^. It was estimated that Mtb accounted for ten million new TB cases and about two million deaths worldwide, and World Health Organization (WHO) declared TB a global emergency. The increasing prevalence of drug-resistant Mtb has further accentuated the need for novel antimycobacterial drugs^3,4^. RNA processing and degradation are required cellular processes that profoundly affect bacterial RNA steady-state levels, which have been recently recognized as potential new targets for novel antimicrobial drug discovery^5–7^.

RNA degradation is mainly executed by a highly structured protein complex named RNA degradosome, comprising ribonucleases (RNases), RNA helicases, and glycolytic enzymes^8^. RNase E, one member of RNases in *E. coli*, is responsible for initial rate-limiting endonucleolytic cleavage in mRNA degradation within an AU-rich single-stranded region and functions as a scaffold for RNA degradosome assembly^9,10^. However, the RNase E gene is not present in most gram-positive bacteria. For instance, *B. subtilis* contains a bifunctional 5′−3′ exo/endoribonuclease RNase J and an RNase Y, essential ribonucleases in global mRNA decay^11–13^. Unlike these organisms, *M. tuberculosis* contains an RNase J and an RNase E (Supplementary Table 1)^14^. Furthermore, the protein sequence identities of RNase J between Mtb and *B. subtilis* or *T. thermophilus* are around 37% and 38%, respectively. Recently, the polynucleotide phosphorylase (PNPase) and ATP-dependent RNA helicase (RhlE) were also identified as major components of the Mtb degradosome via proteomic and transcriptomic screening^15^. Also, RNase J has been reported to be a clinically prevalent mutation in Mtb-mediated multidrug tolerance, exhibiting a significant association with drug resistance^16,17^. According to sequence analysis, RNase J belongs to the β-CASP family of zinc-dependent metallo-β-lactamases^18^. This superfamily of lactamases has a broad substrate spectrum for which there are currently no clinically available inhibitors, having been considered a severe problem for antibiotic therapy in TB^19,20^. Also, transcriptome studies revealed that RNase J1-depleted strains in *B. subtilis* showed an altered abundance for a wide range of mRNA transcripts, accounting for 30% of the genome^21^. Furthermore, strains lacking RNase J1 formed long filaments and were quite sensitive to a wide range of antibiotics and extreme temperatures, suggesting defects in the cell envelope^21,22^.

The crystal structures of RNase J homologs from *B. subtilis*, *T. thermophilus*, and *D. radiodurans* have previously been determined^13,23,24^. RNase J mutants from *T. thermophilus* and *D. radiodurans* lacking the C-terminal domain exist as monomers on size exclusion chromatography, whereas all reported full-length proteins behave as homodimers^23,24^. The C-terminal domain of RNase J from *B. subtilis* and *T. thermophilus* was reported to be required for nuclease activity and protein oligomerization^13,24,25^. However, no structural information is available for Mtb-RNase J, which tremendously dampens understanding of its mechanism in RNA metabolism.

Here, we reported the crystal structures of the apo Mtb-RNase J and its complex with 7-nt RNA with resolutions of 2.445 Å and 3.2 Å, respectively. Mtb-RNase J used zinc ions for two-metal-ion catalysis, forming an active pocket with residues Asp81, Asp85, His86, His148, His170, and His397. Furthermore, the metal ion Mn^2+^ dramatically enhanced the nuclease activity of Mtb-RNase J by increasing its affinity to RNA substrates. Interestingly, Mtb-RNase J formed the active homodimer through the interactions between the β-CASP domain and the β-lactamase domain. Also, the knockout of RNase J dramatically changed the expression levels of the RNA metabolic pathway-related genes.

## Results

### Mtb-RNase J exhibits β-lactamase and ribonuclease activities

**Fig. 1:**
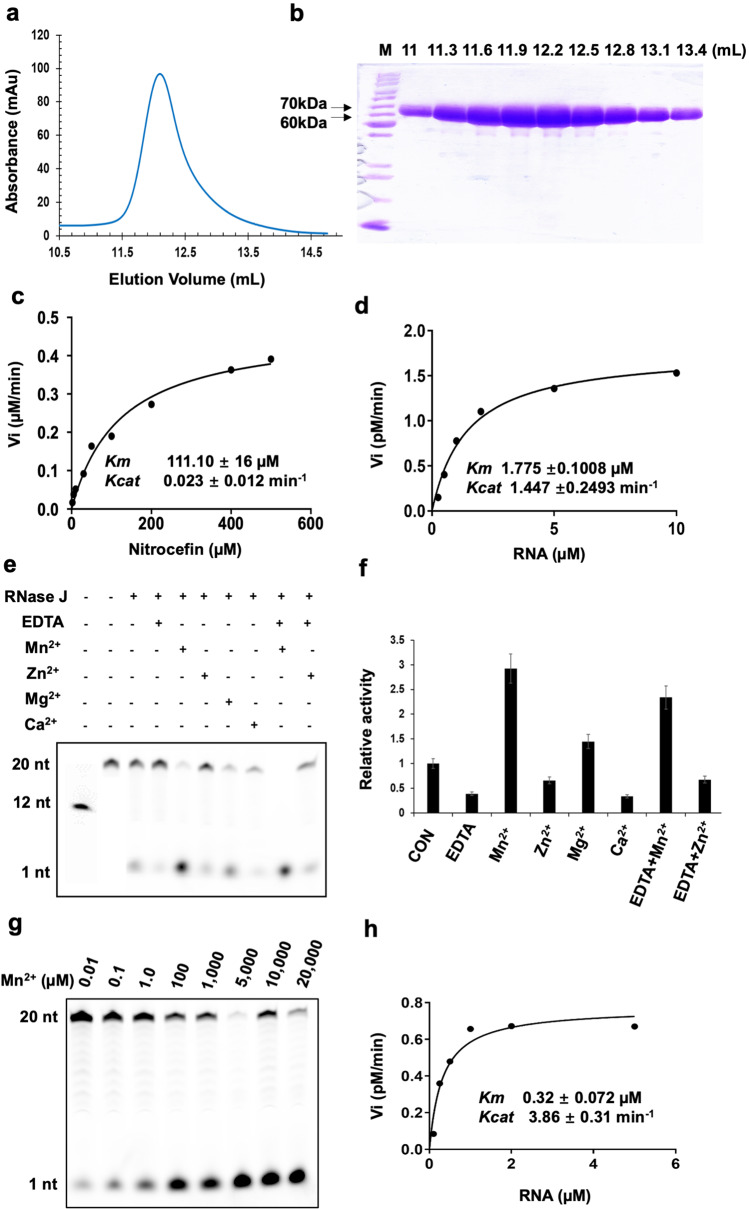
Biochemical characterization of Mtb-RNase J. **a** Gel filtration profile of full-length Mtb-RNase J on a Superdex200 16/300 column. **b** SDS-PAGE result of the corresponding fractions in (**a**). **c** The β-lactamase activity of Mtb-RNase J with the substrate nitrocefin. **d** The ribonuclease activity of Mtb-RNase J with 5′-FAM labeled 20-nt poly(U) RNA as the substrate. **e**, **f**. Different divalent ions affected the ribonuclease activity of Mtb-RNase J. The concentrations of divalent metal ions and EDTA were 5 mM and 10 mM, respectively. 5′-FAM labeled 20-nt poly(U) RNA was used as the substrate at a concentration of 5 µM. CON: without additional ions; EDTA + Mn^2+^: chelated with EDTA, then supplemented with Mn^2+^; EDTA + Zn^2+^: chelated with EDTA, then supplemented with Zn^2+^. **g** Effect of Mn^2+^ to Mtb-RNase J ribonuclease activity with 20-nt poly(U) RNA as the substrate. **h** The ribonuclease activity of Mtb-RNase with 20-nt poly(U) RNA as substrate in the presence of 5 mM Mn^2+^.

To investigate the physiological function, Mtb-RNase J was expressed in *E. coli* Rosetta (DE3) cells and further purified with different chromatography as previously described^26^. The Mtb-RNase J protein was eluted out with a peak at 12.1 mL on the Superdex200 10/300 column, corresponding to an apparent molecular weight (MW) of about 120 kDa (Fig. 1a, b). As the theoretical MW of Mtb-RNase J is around 61 kDa, it indicated Mtb-RNase J was a dimer in solution, which is essential for the nuclease activity as previously reported^23,24^. Moreover, the native page gel and dynamic lighting scattering (DLS) analysis indicated that Mtb-RNase J was highly homogenous (Supplementary Fig. 1a-1b).

Next, the β-lactam ring-contained nitrocefin was used to investigate the β-lactamase activity of Mtb-RNase J. Compared to the dominant β-lactamase BlaC, which acted on nitrocefin with a kinetic parameter *Km* 57 µM/*Kcat* 6670 min^−1^^27^, Mtb-RNase J exhibited a one-fold increase in the *Km* value while a lower *Kcat* value. Its lower substrate affinity and relatively low reactivity probably indicate limited protective activity against β-lactam antibiotics (Fig. 1c and Supplementary Fig. 1c). In addition, as BlaC can be inactivated by EDTA or clavulanate, there are no clinically available inhibitors for the metallo-β-lactamases^27,28^, structural understanding of metallo-β-lactamases will provide vital insights for developing effective inhibitors. Mtb-RNase J has a typical exo/endonuclease activity with mRNA substrates, which is absent in BlaC. However, whether this activity is relevant to bacterial physiology is still unclear.

To further investigate whether Mtb-RNase J has exonuclease activity, a synthetic 5′-end FAM-labeled 20-nt poly(U) single-strand RNA (ssRNA) was employed as the substrate^23^. The results showed that Mtb-RNase J effectively exhibited exonuclease activity (Fig. 1d). In contrast, it did not have a high endonuclease activity toward 5′-end FAM-labeled ssRNA (Fig. 1e, g). According to the gels of 5′ labeled RNA, limited labeled non-mono-nucleotide products have been detected. Furthermore, we used 3′FAM RNA to evaluate the endo activity. Still, limited labeled non-mono-nucleotides products have been detected (Supplementary Fig. 1e). As previous reports showed that the metal ion is critical for the RNase J activity in other species^23,24^, we performed the same assay with a chelation agent EDTA to identify how metal ions work in Mtb-RNase J. It turned out that zinc and manganese functioned in Mtb-RNase J catalysis, while EDTA only decreased the exonuclease reactivity by 60% (Fig. 1e, f). Next, we investigated the influence of different divalent ions, including Zn^2+^, Mn^2+^, Mg^2+^, and Ca^2+^, on the exonuclease activity of Mtb-RNase J. Compared to the enzyme without metal ions, 10 mM Mn^2+^ and Zn^2+^ could restore the FAM-UMP yield by about two-fold and 44% after EDTA chelation. Also, a high concentration of Zn^2+^ could inhibit the enzyme activity of Mtb-RNase J (Supplementary Fig. 1f). In contrast, low concentration Zn^2+^ improved its activity even after being deprived by a chelating agent, phenanthroline (Phen) (Supplementary Fig. 1g). Therefore, Mtb-RNase J preferred low-concentration metal ions for its exonuclease activity. The reactivity of Mtb-RNase J towards unmarked RNA is much faster than that of 5′-FAM-labeled RNA, which indicates that the 5′- FAM molecule has sterical hindrance (Supplementary Fig. 1h). However, it is not easy to measure the in vitro kinetics because of its fast reactivity. Though FAM-tagged RNA does not exist in vivo, its lower reactivity makes it possible to compare the kinetic difference between apo- and metal-ion-added proteins. The kinetic parameters of Mtb-RNase J towards the FAM-labeled 20-nt RNA were determined with 5 mM Mn^2+^ (Fig. 1g, h). The addition of Mn^2+^ exhibited a 4.5-fold decrease in *Km* value and a 1.7-fold increase in *Kcat* value than the protein without metal ion (Fig. 1h and Supplementary Fig. 1d). In addition, the electrophoretic mobility shift assay (EMSA) showed that manganese ions significantly promoted the nucleic acid binding ability of Mtb-RNase J (Supplementary Fig. 1i, j). Thus, Mn^2+^ might enhance the exonuclease activity by improving the substrate affinity of Mtb-RNase J.

Next, to investigate the substrate specificity of Mtb-RNase J, EMSA was employed using the synthetic 5′-end FAM-labeled single-strand RNA as substrates. 0.5 µM poly(A) RNA with different chain lengths was incubated with 0, 0.1, 0.5, 1, and 10 µM Mtb-RNase J (D85A), a catalytically inactive mutant. Results showed that full-length Mtb-RNase J preferred 9-nt RNA while exhibiting a low binding affinity towards 5-nt or 12-nt RNA substrates (Supplementary Fig. 2). We speculated that the substrate chain-length selectivity resulted from the substrate tunnel in Mtb-RNase J, implying that the appropriate chain-length RNA might facilitate the necessary interaction with the protein.

### The overall structure of Mtb-RNase J

**Fig. 2:**
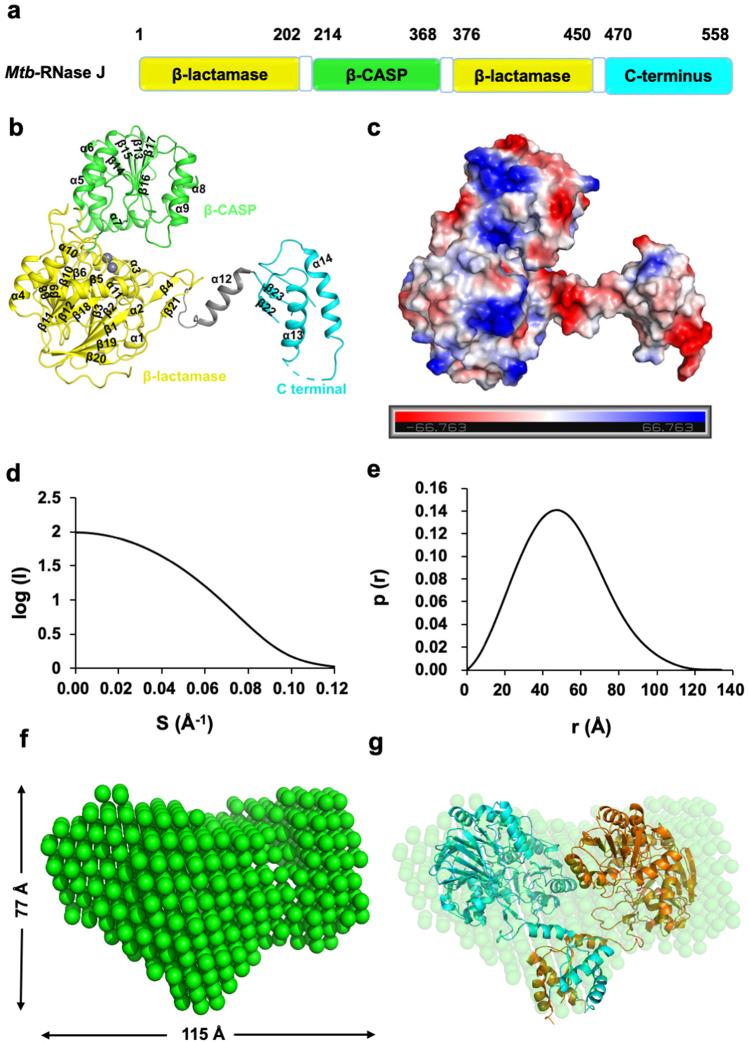
The overall structure of Mtb-RNase J. **a** Domain structure of Mtb-RNase J. The β-lactamase domain, β-CASP domain, and the C-terminus are colored in yellow, green, and cyan, respectively. **b** Ribbon representation of Mtb-RNase J, showing 14 α-helices and 23 β-sheets, with corresponding colors in (**a**). The zinc ions are shown in gray spheres. **c** The electrostatic potential surface of Mtb-RNase J. Red: negative potential; blue: positive potential. **d** The experimental scattering curve of Mtb-RNase J. **e**. The distance distribution function curve of Mtb-RNase J. **f** SAXS solution structure of Mtb-RNase J. **g** The crystal structure of Mtb-RNase J was fitted into the ab initio envelope obtained from SAXS. Cyan and orange represent a monomer of the dimeric Mtb-RNase J, respectively.

**Fig. 3:**
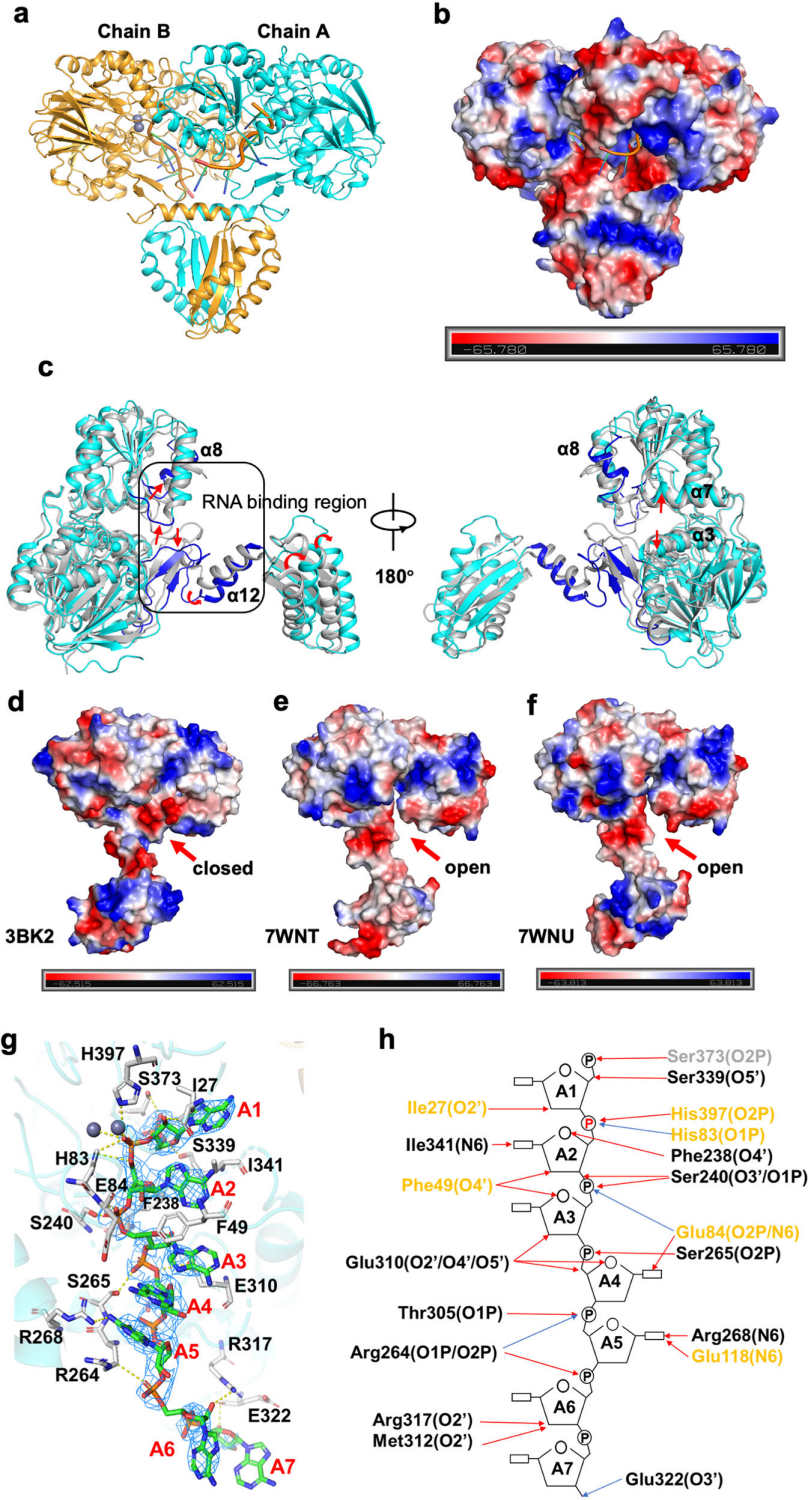
Conformational changes of Mtb-RNase J upon RNA binding. **a** Overall structure of the Mtb-RNase J/ssRNA complex. The ssRNA was shown in a stick model. The metal ions were shown in gray spheres. Chains A and B were colored blue and orange, respectively. **b** The electrostatic potential surface of the Mtb-RNase J/ssRNA complex. Red: negative potential; blue: positive potential. **c** Superimposition of the Tth-RNase J and Mtb-RNase J structures. Mtb-RNase J and Tth-RNase J were shown in cyan and gray, respectively. The significant differences in RNA binding were highlighted in blue color. The red arrows show the shifts. A black box represented the RNA binding region. **d**–**f** The electrostatic potential surfaces of Tth-RNase J (**d**, PDB: 3BK2), apo-Mtb-RNase J (**e**, PDB: 7WNT), and chain B of Mtb-RNase J/ssRNA complex (**f**, PDB: 7WNU) were shown, respectively. The red arrows denoted the different conformations of RNase J. **g** Details of the RNA binding sites in the Mtb-RNase J/ssRNA complex. RNase J was shown in a cartoon model, whereas ssRNA was in a stick model with an electron density map contoured to 1.0 *σ* at the 2Fo-Fc map. The slice of the structure was viewed from the inside of the protein looking outwards. **h** The schematic representation of the RNA binding sites. Residues involved in RNA-binding on the β-lactamases, the β-CASP, and the loop were shown in yellow, black, and gray, respectively. Blue and red arrows indicated interactions through the main and the side chains of different residues, respectively. The scissile phosphate was highlighted in red.

To reveal the structural basis of Mtb-RNase J in RNA metabolism, we solved the crystal structures of wild-type Mtb-RNase J and its inactive catalytic mutant (D85A) in complex with a synthesized 7-nt poly(A) ssRNA (denoted as Mtb-RNase J/ssRNA). The apo structure contained one molecule in the asymmetric unit, while the complex structure contained two molecules in the asymmetric unit, named chains A and B, respectively (Figs. 2b and 3a). The Mtb-RNase J contained fourteen α-helix, eight 3_10_ helices, and twenty-four β-stands, comprising three distinct globular domains, including an N-terminal β-lactamase domain (residues 5–205 and 376–450), an inserted β-CASP domain (residues 206–375) and a C-terminal domain (residues 471–553) (Fig. 2a, b). Moreover, the β-lactamase domain contained double opposing seven-stranded β-sheets sandwiched by three α-helices on either side, a characteristic fold of the RNase J superfamily protein (Supplementary Fig. 3)^29^. The β-CASP domain was formed by five parallel β-sheets surrounded by three α-helices on each side. Also, the C-terminal domain of Mtb-RNase J was distant from the catalytic core and was connected to the β-lactamase domain by an extended linker helix (residues 456–470), which consisted of a two-stranded β sheet facing two helices (Fig. 2b). The surface electrostatic potential of Mtb-RNase J was evenly distributed, except that the RNA binding pocket was full of positive charges (Fig. 2c).

Furthermore, we investigated the solution status of the Mtb-RNase J by the small-angle X-ray scattering (SAXS) method (Fig. 2d, e). The Mtb-RNase J behaved well in the solution, and the maximum dimension (Dmax) from the distance distribution function p(r) was around 120 Å (Fig. 2e). Moreover, when superimposed, the crystal structure with the ab initio envelope obtained from SAXS, Mtb-RNase J showed high similarities, confirming that the purified Mtb-RNase J in vitro was indeed the active dimeric form (Fig. 2d–g).

### Conformational change upon RNA binding

The Mtb-RNase J/ssRNA complex structure was a homodimer, with each chain coupling with one RNA located at the interface of the β-lactamase and β-CASP domains (Fig. 3a). Furthermore, the overall surface electrostatic potential was evenly distributed, whereas the RNA binding area was full of positive charges (Fig. 3b). The Tth-RNase J structure revealed that the catalytic cleft (located in the deep cleft between β-lactamase and β-CASP domain) would be widened to create a substrate channel upon RNA binding^30^. However, superimposing the Mtb-RNase J apo structure with RNase J/ssRNA complex revealed similar features of overall folds, which had high similarities among the 438 Cα atoms with an RMSD value of 0.468 Å (Supplementary Fig. 4a, b). In addition, no significant conformational changes in the protein backbones were observed in the catalytic cleft. The structural differences existed mainly in the C-terminal region, where the α13 and α14 shifted away when binding with ssRNA (Supplementary Fig. 4a–d). Notably, the side chains of a couple of residues dramatically changed to facilitate RNA binding (Supplementary Fig. 4e).

We further superimposed the Mtb-RNase J/ssRNA complex (PDB: 7WNU) with Tth-RNase J/RNA (PDB: 3T3N) and its RNA-free form (PDB: 3BK2)^28^. The resulting RMSD values were 1.336 Å and 2.129 Å, respectively. Compared with the closed conformation in the apo Tth-RNase J, significant conformational changes occurred around the catalytic cleft of both the apo and RNA-binding Mtb-RNase J, resembling the open conformations of that in the Tth-RNase J/RNA structure (Fig. 3c–f). The loop (residues 305–313) between β15 and α8 moved away from α8 (residues 314–320), shifting about 17° towards RNA to facilitate substrate binding, which would probably bring residues Arg317, Thr305, Thr307, and E310 into the contact with the nucleotides. In addition, the two β-strands, β4 (residues 58–61) and β21 (residues 447–449), and the loops around them at the entrance of the substrate channel underwent relatively large displacement among the open and closed conformations (Fig. 3c). Thus, we suspected these two β-strands were closely related to the RNA binding. Furthermore, residue Asp52 formed polar contacts with the fourth nucleotide in Tth-RNase J, but was displaced by as much as 3.1 Å away from the substrate tunnel in Mtb-RNase J, which might result in the loss of the polar contacts (Supplementary Fig. 4c, d).

Multiple molecular interactions were generated to stabilize the substrate upon RNA binding (Fig. 3g, h). The first phosphate was stabilized by the side chains of Ile27, His375, Ser339, Ser373, and Gly374. The second phosphate directly interacted with the two zinc ions. Also, the side chain of His397 and the backbone of His83 interacted with the scissile phosphates OP2 and OP1 (Fig. 3g, h). Glu84 and Ser240 formed hydrogen bonds with the third phosphate to stabilize RNA binding. In addition, Glu310 formed polar contacts with the O2 atom of the third AMP (Supplementary Fig. 4e). Ser265 significantly facilitated the rest two AMPs through multiple interactions with their phosphates. Furthermore, Glu118 and Thr305 bound with the fifth AMP at the substrate entrance pocket through their side chains (Fig. 3g, h).

Although the RNA conformations were almost identical in chains A and B, molecular interactions exhibited differences (Supplementary Fig. 4f). For example, the residues His375, Ser339, Ser240, Glu310, and Glu118 in chain B dramatically changed the side-chain orientations. Meanwhile, new contacts were generated between the fifth AMP and the side chains of Thr305 and Ala314 (Supplementary Fig. 4f). The results probably represented different states during substrate binding, as chain A might reflect the status of the catalytic reaction, while chain B might refer to the intermediate state of the RNA binding process.

### The active sites of Mtb-RNase J

**Fig. 4:**
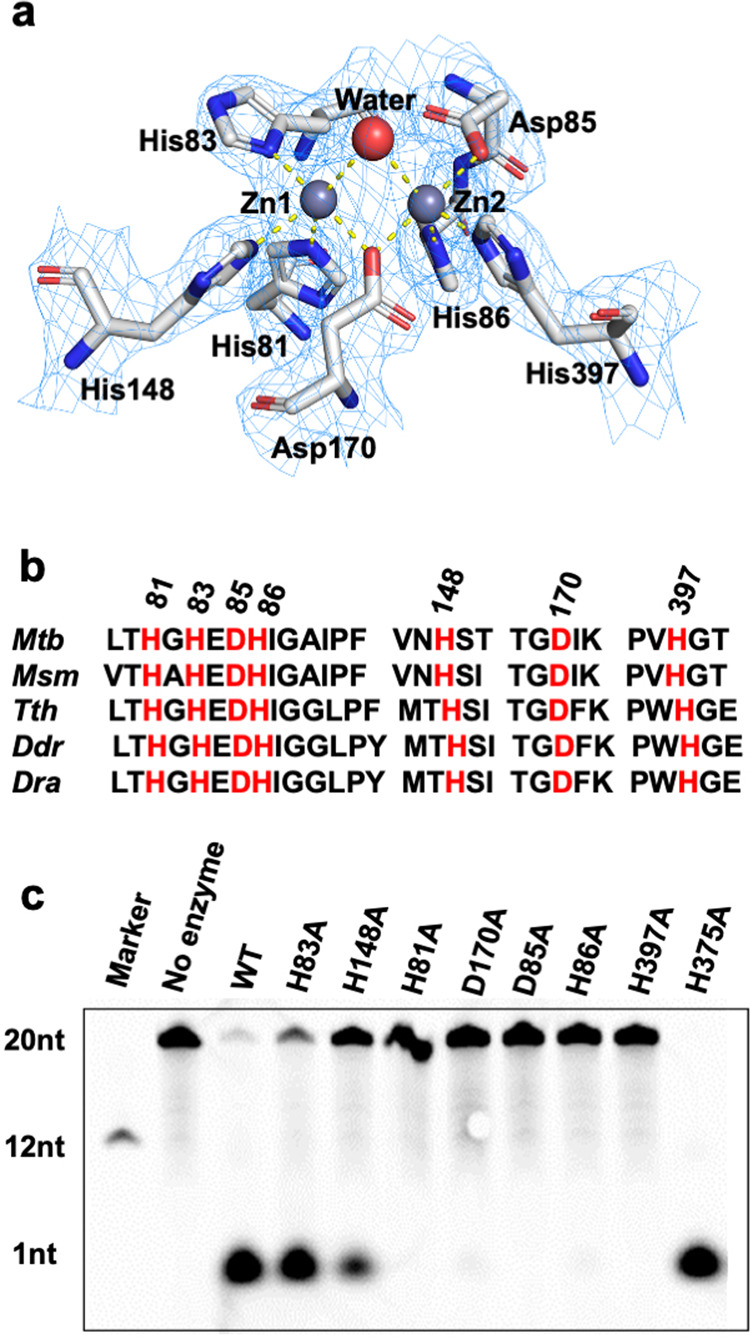
The active sites of Mtb-RNase J. **a** The active sites of Mtb-RNase J. Residues involved in metal chelation were shown as stick models. The Zn^2+^ were shown as gray spheres. Yellow dashed lines indicated Zn^2+^ coordination. The bridging water was shown as a red sphere. The electron density map was contoured to 2.0 *σ*. **b** Sequence alignment of the active sites among different RNase J proteins: Mtb (*Mycobacterium tuberculosis*), Msm (*Mycobacterium smegmatis*), Tth (*Thermus thermophilus*), Bsu (*Bacillus subtilis*), Ddr (*Deinococcus deserti*), and Dra (*Deinococcus radiodurans*). The conserved binding sites are shown in red. **c** The ribonuclease activities of Mtb-RNase J and mutants were assayed with a 20-nt ssRNA as the substrate.

The active sites of Mtb-RNase J were located in the deep cleft between the β-lactamase and the β-CASP domains, consisting of double octahedrally coordinated Zn^2+^ ions, similar to those previously reported (Fig. 4a)^13^. No zinc ion was added during the crystallization process; however, apparent electron densities for double Zn^2+^ were observed in the active sites of Mtb-RNase J (Fig. 4a). Furthermore, inductive-coupled plasma mass spectrometry (ICP-MS) assay and X-ray fluorescence scattering analysis with RNase J crystals confirmed that Zn^2+^ was present in purified Mtb-RNase J protein and in crystals, evidenced by a strong peak at the ZnK-Edge (Supplementary Fig. 5). In addition, a water molecule was found in the active center to bridge both zinc ions to form hydrogen bonds with the side chains of Asp85 and His83 (Fig. 4a), resembling the characteristic two-zinc ion catalytic center of the homologous RNase J^23,24^. In Mtb-RNase J, each zinc ion was bound in a typical octahedral configuration. One zinc atom (named Zn-1) was coordinated by residues His81, His83, and His148, while the other (named Zn-2) interacted with residues Asp85, His86, and His397 (Fig. 4a). Sequence alignment indicated that these coordinated residues were highly conserved in the RNase J family (Fig. 4b). In addition, Ala substitutions of these residues severely inhibited the nuclease activity of Mtb-RNase J (Fig. 4c). The nuclease activities of Mtb RNase J were almost lost when the Zn-2-coordinated residues were mutated; however, the RNase J retained activities when Zn-1-coordinated residues His83 and His148 were mutated (Fig. 4c). The results showed that the exact coordination of Zn-2 was necessary for the enzyme-catalyzed reaction, whereas Zn-1 was a constituent ion, most likely stabilizing the active center structure in the catalytic process.

### The dimeric mechanism of Mtb-RNase J

**Fig. 5:**
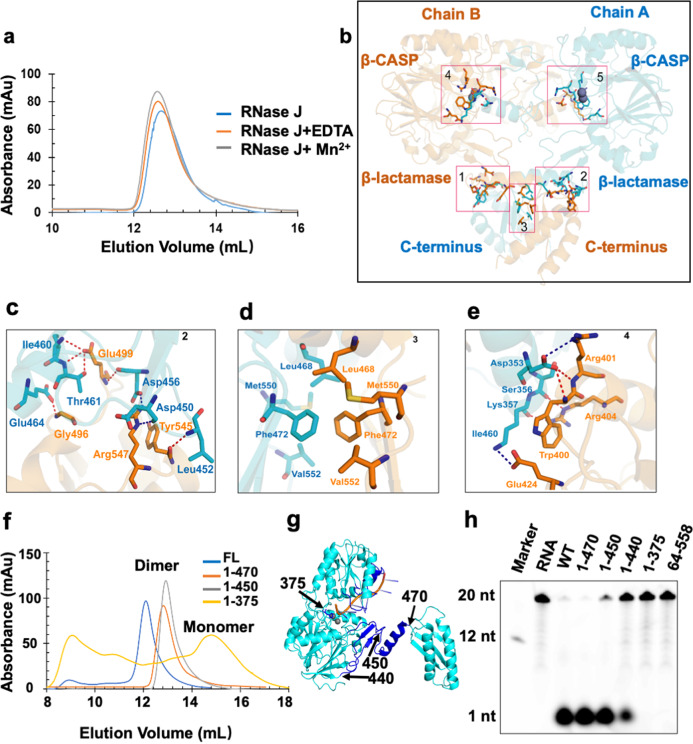
The dimeric mechanism of Mtb-RNase J. **a** Superimposition of the gel filtration profiles of Mtb-RNase J alone or with 10 mM EDTA or Mn^2+^ on a Superdex200 10/300 column. **b** Overview of the interacting interfaces of the two Mtb-RNase J chains. Chains A and B were colored cyan and orange, respectively. The detailed residues were shown in the sticks. **c**–**e** Enlarged view of the interacting interface, corresponding to (**b**). The detailed residues were shown in stick models. **f** Gel filtration profiles of Mtb-RNase J and its different truncations on a Superdex200 10/300 column. **g** Ribbon representation of Mtb-RNase J/ssRNA structure, with different numbers denoting the truncated sites. **h** The ribonuclease activities of wild type (WT) and different truncations of Mtb-RNase J.

The divalent cations at the dimerization interface facilitated protein dimerization in Dra-RNase J by coordinating with residues (Gly59, Asp61, and Asp456) from chain A and residues (Gln476 and Glu477) from chain B, respectively (Fig. S6)^23,24^. However, the solution status of Mtb-RNase J did not change when EDTA or the metal ion Mn^2+^ were added, different from that in Dra-RNase J (Fig. 5a). Moreover, no corresponding divalent cations were observed in the crystal structures of Mtb-RNase J and Tth-RNase J, indicating that the role of divalent ions in homodimerization is not universal (Fig. 5b). Furthermore, residues Gly56, Asp58, and Asp450 of Mtb-RNase J were relatively conserved among different species, whereas Ser469 and Ser470 were not (Supplementary Fig. 6a-6b). Therefore, no available coordinated residues existed for divalent ions binding, indicating Mtb-RNase J might have a different dimerization mechanism (Supplementary Fig. 6). Also, Mtb-RNase J adopted a featured dimeric structure, alongside with the C-terminus crossed (Fig. 3a, 5b). Although studies indicated that the oligomerization of RNase J was crucial for nuclease activity and was supposed to be mainly mediated by the C-terminal domain of the RNase J^27,28,31^, the C-terminal truncated mutants of Mtb-RNase J still appeared to be dimeric in solution, indicating the dimeric mechanism was probably different from other homologs (Fig. 5f). Thus, the C-terminal domain of Mtb-RNase J may resemble the equivalent domain of RNase E, which functions as the scaffold for degradosome assembly^10^.

The structural architectures of the two chains in Mtb-RNase J were identical, and the interacted regions were located in the swapped C-terminal domain (Fig. 5b). The multiple polar residues from chain A formed contacts with chain B, which probably stabilized the dimeric state of Mtb-RNase J (Supplementary Table 2). For example, Arg547 interacted with residues Asp450 and Asp456. Tyr 545 formed hydrogen bonds with the nitrogen atom of Leu452. Also, the side-chain oxygen of Glu499 was involved in direct hydrogen-bond interactions with the backbone nitrogen of Ile460 and the side-chain oxygen of Thr461 with distances of 3.2 Å and 2.9 Å, respectively. Moreover, Gly496 formed a hydrogen bond with the side chain of Glu464 at a distance of 2.36 Å (Fig. 5c). In addition, a small hydrophobic cleft was formed by the side chains of Val552, Met550, Phe472, and Leu468 that were located on the surface of the C-terminal β sheet (Fig. 5d). Furthermore, the truncated mutants (1-450 and 1-470) still appeared to be dimeric in solution, resembling the property of full-length protein (Fig. 5f). Thus, the molecular interactions in the C-terminal domain above were not essential for Mtb-RNase J oligomerization.

In the dimeric interface, residues showed significant interactions between the β-CASP and the β-lactamase domain (Fig. 5e and Supplementary Table 3). For instance, Glu424 and Arg401 interacted with Lys357 and Asp353, respectively. In addition, Asp404 formed hydrogen bonds with the backbone oxygen atoms of Lys357 and Ser356 (Fig. 5e and Supplementary Table 3). To further investigate the effects of these interactions on Mtb-RNase J oligomerization, we constructed a truncated mutant 1-375, and found it existed from a monomer to high oligomer in the gel-filtration chromatography, which might result from the unstable truncated protein (Fig. 5f). Surprisingly, this interface was also closely located near the exit of the RNA binding channel. The β-CASP and the β-lactamase domain (from the other chain) formed a deep and compact cleft, large enough to accommodate RNA molecules. The cleft included the loops R336-344 and R367-378 in chain A, and loops R24-30, R44-54, and the α9 helix in chain B (Figs. 5g, h). The enzymatic assay further confirmed that the monomeric mutant (1-375) exhibited little activity towards RNA substrates, whereas the C-terminal domain-depletion protein (1-470) exhibited a competitive activity compared to the full-length protein (Fig. 5h). This indicated that the Mtb-RNase J’s C-terminal domain was neither essential for protein dimerization nor RNase activity maintenance.

**Fig. 6:**
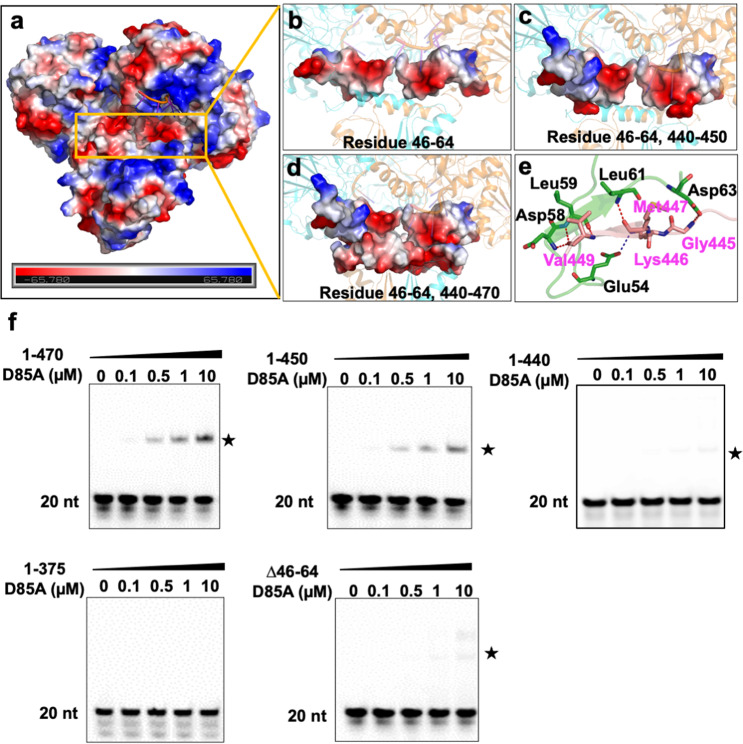
Negatively charged regions 46-64 and 440-450 of Mtb-RNase J were crucial for RNA binding. **a** The overall electrostatic distribution of the Mtb-RNase J/ssRNA complex. The yellow box represented the substrate-entrance pocket. Red: negative potential; blue: positive potential. **b** The surface electrostatic potential of region 46-64 in Mtb-RNase J. **c** The surface electrostatic potentials of regions 46–64 and 440–450 in Mtb-RNase J. **d** The surface electrostatic potentials of regions 46–64 and 440–470 in Mtb-RNase J. **e**. The interaction between regions 46–64 and 440–450. The residues were shown in stick models. **f** The EMSA result of Mtb-RNase J (D85A) and truncated mutants with 0.5 μM 20-nt poly (U) RNA. The asterisk indicated the bound RNA.

Compared with the Mtb-RNase J/ssRNA complex, the cleft in Mtb-RNase J was almost perpendicular to the RNA molecule, generating two opposite orientations for the RNA channel exit from the catalytic center to the surface of the protein (Fig. 6a and Supplementary Fig. 4). One orientation towards loop R367-378 was somewhat positively charged, while the other was negatively charged. Considering the charge characteristic of an RNA molecule, we speculated that the former orientation was the rest RNA binding tunnel in the nuclease reaction. However, the lack of binding sites for RNA products in the exonuclease reaction indicated that the release of the 5′ proximal product was too fast to be tracked (Fig. 6a and Supplementary Fig. 4).

### Negatively charged peptides 46–64 and 440–450 are crucial for RNA binding

Truncation (1–440) severely impaired the ribonuclease activity of Mtb-RNase J, despite that the C-terminal truncation (1–470) exhibited a competitive activity with the full-length protein (Fig. 5h). However, the truncated protein (1–450) displayed a slightly decreased activity, suggesting the fragment (440–450) might function in dimerization and ribonuclease activity of Mtb-RNase J (Fig. 5h). Also, the fragment (440–450) in the Mtb-RNase J/ssRNA complex formed multiple polar contacts with the highly negative-charged fragment (46–64) located near the substrate entrance (Fig. 6a–e). Furthermore, the truncation (64–558) also abolished the ribonuclease activity of Mtb-RNase J (Fig. 5g, h).

Thus, these negative-charged fragments (46–64, 440–450, and 451–470) might generate a robust polar environment with the positive-charged RNA-bound tunnel, which facilitates the quick recognition of RNA substrates through electronic interactions (Fig. 6a–d). The hypothesis was verified using FAM-labeled 20 nt RNA as substrates to assay the RNA binding ability. Results showed that mutant 1-375/D85A abolished the RNA binding ability, whereas mutants △46-64/D85A and 1-440/D85A retained little RNA binding capacity (Fig. 6f). Also, mutant 1-450/D85A displayed slightly reduced RNA binding ability compared to mutant 1-470/D85A (Fig. 6f). All the above data suggested that negatively charged fragments (Gly46-Met64 and Gly440-Asp450) could regulate the ribonuclease activity of Mtb-RNase J by affecting its RNA binding capacity.

### RNase J alters the growth and morphology of *Mycobacterium smegmatis* by affecting cell wall synthesis and lipid metabolism

**Fig. 7:**
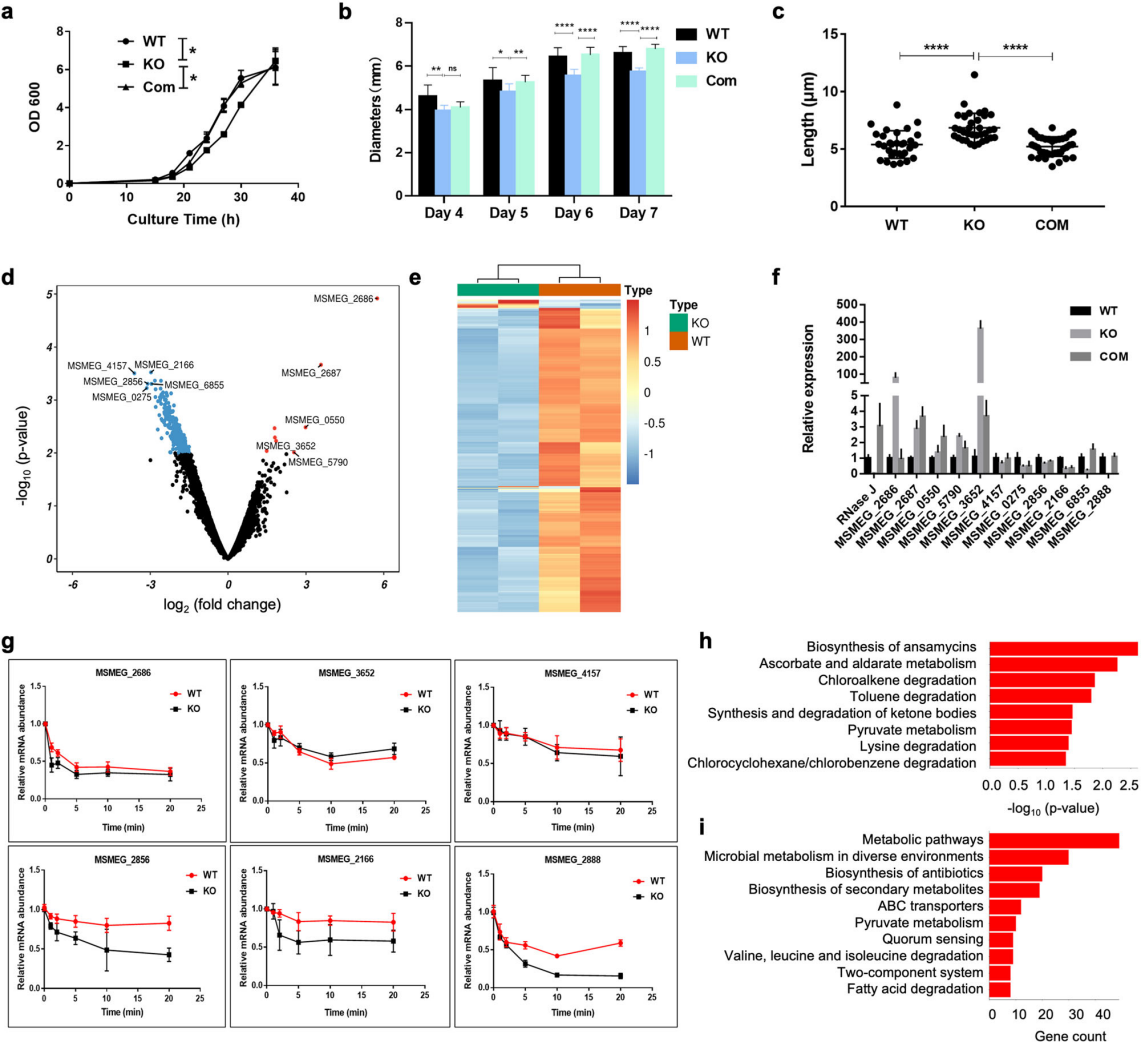
RNase J alters the growth and morphology of *Mycobacterium smegmatis* by affecting cell wall synthesis and lipid metabolism. **a** The growth curve of wild-type (WT), RNase J KO, and RNase J complemented (COM) Msm strains. **b** The colony size difference among WT, KO, and COM Msm strains. **c** Cell lengths of WT, KO, and COM strains with transmission electron microscopy (TEM) observation. **d** Volcano plot of differentially expressed genes between WT RNase J and its KO strain by RNA-seq analysis. The significant differentially expressed genes were labeled by the standard of pval<0.05&|log2FC | >1, up as red, down as blue. **e** Heat map of significant differentially expressed genes between WT RNase J and its KO strain. The standard was shown as in (**d**). **f** The expression levels of twelve significant differential genes among the three strains were detected by the Q-PCR method. **g** Influence of rifampin on the mRNA stability of six represented genes in the WT or KO Msm strains. **h**, **i** The KEGG annotation analysis revealed that 49 genes in the metabolic pathway were significantly differentially expressed between WT RNase J and its knockout strain.

The putative RNases presented in Mtb and *M. smegmatis* (Msm) are the same. The RNase J homologs share high sequence identity and similarity (86% and 92%, respectively) (Supplementary Fig. 3 and Supplementary Table 1). Msm, one surrogate strain of Mtb, has already been employed to understand mycobacterial physiology and metabolism^32^. To investigate the physiological function of RNase J, we constructed the RNase J knockout (KO) strain in Msm by the homologous recombination method (Supplementary Fig. 7). Compared to the wild-type (WT) strain of Msm, the RNase J KO strain showed a slower growth rate in the liquid medium, whereas the growth was recovered after the complement of the RNase J gene (Fig. 7a). In addition, the plate colony sizes of the KO strain were significantly smaller, and the mycobacterial lengths were longer than the WT strain, evidenced by the electron microscopy (EM) observation (Fig. 7a–c and Supplementary Fig. 8). Also, the colony surface of the KO strain was drier and more wrinkled than the WT strain. The morphological difference may be because RNase J knockout affects cell wall synthesis and lipid metabolism in *Mycobacterium smegmatis* (Supplementary Fig. 8).

To further identify the RNase J function at the transcriptome level, the WT and RNase J KO strains of Msm were performed with bulk-RNA sequencing (RNA-seq). We found that 349 differentially expressed genes (DEGs) existed in the KO strain compared with the WT one, among which eight genes were significantly up-regulated and 341 were significantly down-regulated with *p* < 0.05&|log2FC| >1 as the critical value (Fig. 7d, e). Next, the expression levels of twelve significant differential genes among the three strains were confirmed by the quantitative-PCR method (Fig. 7f). Also, the rifampin time-course experiment showed that the mRNA stability of three genes (Msmeg_2856, Msmeg_2166, and Msmeg_2888) was affected in the RNase J mutant, whereas the others were not affected (Fig. 7g). KEGG enrichment analysis revealed that the DEGs were mainly enriched in metabolic pathways, microbial metabolism in diverse environments, antibiotics biosynthesis, and secondary metabolites biosynthesis (Fig. 7h, i). In particular, genes related to the cell membrane and cell wall were significantly regulated (Fig. 7h, i). For example, the microcompartment protein family gene MSMEG_0275 and the virulence factor Mce family gene MSMEG_2856 were significantly down-regulated, while a hypothetical transmembrane protein MSMEG_2686 and the phospholipid biosynthesis-related CDP-diacylglycerol-glycerol-3-phosphate 3-phosphatidyl transferase MSMEG_3652 were significantly up-regulated (Fig. 7d). Thus, the results suggest that RNase J may alter the growth and morphology of *Mycobacterium* by affecting cell wall synthesis and lipid metabolism.

## Discussion

mRNA degradation is an essential process of post-transcriptional regulation in cells^33^. Bacteria regulate the expression of specific genes through this process to quickly adapt to the growth environment changes. Due to the significant variation of mRNA degradation complexes among different species, it can provide new targets for developing antibacterial drugs^34^. RNase J, a vital component of the Mtb degradation complex, plays an essential role in regulating the growth and survival of bacteria^16,17^. More evidence implied that targeting RNase J might provide one new strategy for treating Mtb infection. Here, we report the apo Mtb-RNase J crystal structures and their complex with 7-nt RNA. Mtb-RNase J can use zinc ions for two-metal-ion catalysis, forming an active pocket with several residues. Moreover, Mn^2+^ could dramatically enhance the nuclease activity of Mtb-RNase J by increasing its affinity to RNA substrates. Mtb-RNase J exhibits β-lactamase and exonuclease activity, making it a potential target for clinical drug development (Fig. 1).

Structural superimposition of Mtb-RNase J with *S. coelicolor* RNase J (PDB ID: 5A0T), *S. epidermidis* RNase J1 (PDB ID: 6K6S), and RNase J2 (PDB ID: 6K6W) revealed overall RMSD values of 1.069 Å, 1.215 Å, and 0.771 Å, respectively, indicating that the β-lactamase and β-CASP domains were highly conserved in different species (Supplementary Fig. 9)^35,36^. Also, the metal-ions-interacting residues in the catalytic pocket were conserved between Mtb-RNase J and *S. coelicolor* RNase J^35^. In addition, Mtb-RNase J exhibited a different 3D structure from RNase AS (PDB ID: 4OKJ), one member of the ribonuclease family, which lacks the CASP and C-terminal domains but acts as a 3′−5′-exoribonuclease and specifically hydrolyzes poly(A) RNA^14,37^. Interestingly, Mtb-RNase J does not form the active homodimer through the C-terminal domain but through the interactions between the β-CASP domain and the β-lactamase domain, different from the homologous RNase J in *T. thermophilus* and *D. radiodurans* (Figs. 2–4)^23,24^. Moreover, in Dra-RNase J, the divalent cation at the dimerization interface facilitates protein dimerization, which is coordinated by residues (Gly59, Asp61, and Asp456) from chain A and residues (Gln476 and Glu477) from chain B, respectively^23,24^. However, no divalent cations were observed in the crystal structure of Mtb-RNase J and Tth-RNase J. Furthermore, the in vitro assay showed that EDTA and Mn^2+^ exhibited no influence on protein dimerization (Fig. 5a). This means that divalent ions’ role in RNase J homodimerization is not universal among different species.

In addition, we found that the RNase J knockout significantly slowed the growth rate and changed the colony morphologies of *Mycobacterium smegmatis*, which was recovered by complementing the RNase J gene (Fig. 7a–c). Furthermore, 349 differentially expressed genes (DEGs) were up-regulated or down-regulated at the transcriptome level in the KO strain (Fig. 7d–i). The Q-PCR and rifampin time-course experiment confirmed that mRNA expression levels of the DEGs were either directly affected by the RNase J mutant or indirectly influenced by some transcription factors (Fig. 7g). Moreover, 49 DEGs were found to be concentrated in metabolic pathways by the KEGG analysis (Fig. 7h, i). Similarly, a homolog of Msm RNase J, Mab_3083c, was reported to be able to affect the colony morphotype, aggregation, and sliding motility in *Mycobacterium abscessus*^38^. In summary, our current work demonstrated the detailed structural and functional basis of Mtb-RNase J, which might provide insights into the treatment of TB.

## Methods

### Strains and plasmid construction

Full-length Mtb-RNase J (Uniprot: P9WGZ9) was amplified by the PCR method from the Mycobacterium tuberculosis H37Rv and was cloned into a modified pET28a vector containing a fused N-terminal 6xHis-sumo tag. Site-directed mutants were constructed according to the standard QuikChange Site-Directed Mutagenesis protocol (Stratagene, USA) using the wild-type Mtb-RNase J as the template. All the constructs were confirmed by DNA sequencing.

### Protein Expression and Purification

Plasmids were transformed into *E. coli* Rosetta (DE3) competent cells for protein expression. *E. coli* cells were cultured in LB medium with 50 µg/mL kanamycin and 34 µg/mL chloramphenicol at 37 °C. Strains containing the expression plasmid were induced at OD_600_ of 0.8 by adding 0.5 mM IPTG, then continuously grown at 16 °C for an additional 20 hours before being harvested at 4 °C. Cells were suspended in the binding buffer (20 mM Tris, pH 8.0, 1 M NaCl, 10 mM imidazole, 10 % glycerol, 2 mM β-ME) and lysed by a high-pressure homogenizer. The lysate was then clarified by centrifugation at 18,000 rpm for one hour at 4 °C. Next, the supernatant was purified by Ni-NTA affinity chromatography. After cleavage by the ULP1 protease, the protein was further purified by the Q-Trap HP column to remove nucleic acids and the sumo-tag. The target protein was finally purified by a Superdex 200 10/300 GL column with a buffer containing 20 mM Tris, pH 8.0, 0.1 M NaCl, and 2 mM DTT.

### Dynamic light scattering (DLS) measurement

The DLS data were collected on the DYNAMICS software from DynaPro NanoStar (Wyatt Technology), operating at a light source wavelength of 658 nm and a fixed scattering angle of 90°. The fresh proteins were diluted to 1 mg/mL with a buffer containing 20 mM Tris-HCl (pH 8.0), 100 mM NaCl, 2 mM TCEP, and 5% glycerol at 25 °C.

### Substrate binding specificity

6-carboxy-fluorescein (FAM)-labeled poly(A) RNA substrates (Huagene Biotech, China) with different lengths were used to investigate the substrate specificity. The standard activity assay was performed in a 10 µL mixture containing 5 µM of RNA substrates and different concentrations of Mtb-RNase J D85A mutant, which lost its RNA degradation activity, in a buffer containing 50 mM Tris-HCl pH 7.4 and 100 mM KCl. Reaction mixtures were incubated on the ice for 15 min and terminated by adding 2 µL 6x loading buffer (0.05% Bromophenol blue, 0.035% Xylene Cyanol FF, 12% Ficoll 400), and then analyzed on a natural 6% polyacrylamide gel. The gels were imaged using a Typhoon FLA 9000 machine. The intensities of the bands were quantified by the ImageQuant TL system.

### The nuclease activity assay

RNAs labeled with 6-carboxyfluorescein at the 5′-end or 3′-end were synthesized by Huagene (Shanghai). For a typical nuclease digestion assay, 5 µM RNA was incubated with various concentrations (1–100 µM) of freshly prepared Mtb-RNase J or mutant proteins in a 10 µl reaction volume containing 50 mM Tris (pH 8.0), 100 mM KCl, 0.1 mg/ml BSA, 1 mM DTT, and 5 mM MnCl_2_ at 30 °C for 10-30 min. The reactions were quenched with a 6 x stop buffer (10 mM EDTA, 98% formamide) and incubated at 95 °C for 10 min. Reaction products were resolved on 15% polyacrylamide sequencing gels containing 7 M urea. To determine the metal ion effects, various kinds of metals, including MgCl_2_ (5 mM), CaCl_2_ (5 mM), MnCl_2_ (5 mM), ZnCl_2_ (5 mM), or EDTA (10 mM), were added to the reaction buffer. The exonuclease activity was tested using the 5′-end FAM-labeled RNA, and the endonuclease activity was tested using the 3′-end FAM-labeled RNA as substrates. The 20-nt RNA sequence was UUCCGUUUAUUCAUACUUCA.

For the unmarked nuclease digestion assay, unmarked 20-nt RNA (sequence, UUCCGUUUAU UCAUACUUCA) and the paired 5′-end DIG labeled-probe (sequence, AAGGCAAAUA AGUAUGAAGU) were synthesized by Huagene (Shanghai). 1 µM Mtb-RNase J was incubated with various concentrations (0.25, 0.5, 1, 2, 5, 10 µM) of unmarked RNA in a 10 µl reaction volume containing 50 mM Tris (pH 8.0), 100 mM KCl, 0.1 mg/mL BSA, and 1 mM DTT at 30 °C for 0.5, 2, 5, 7.5, and 10 min. The reactions were quenched with a 6 x stop buffer (10 mM EDTA, 98% formamide) and incubated at 95 °C for 10 min. Reaction products were resolved on 16% polyacrylamide sequencing gels containing 7 M urea. Probe hybridization and immune-detection procedures are referred to as DIG Luminescent Detection Kit from Roche (Cat. No. 11363514910).

### Nitrocefin hydrolysis assay

Nitrocefin was employed to detect the β-lactamase activity of Mtb-RNase J. The steady-state rate of hydrolysis of the β-lactam ring was monitored as an increase in the absorbance at 486 nm (ε = 20,500 M^−1^ cm^−1^). Assays were performed in a buffer containing 100 mM NaCl and 50 mM Tris pH 7.0. Reactions were initiated by the addition of different concentration substrates (1–800 uM) with 20 µM Mtb-RNase J protein. All kinetic assays were carried out in triplicates at 30 °C. The mutants’ quantifications were performed similarly with the wild-type Mtb-RNase J.

### Crystallization and Data Collection

All crystals were grown by the sitting-drop vapor diffusion method at 18 °C. Purified Mtb-RNase J was diluted to around 10 mg/mL and incubated with a 9-mer poly(A) RNA at a protein-to-RNA ratio of 1:1.2 (mol/mol) in a buffer containing 20 mM Tris-HCl (pH 7.4), 100 mM NaCl. The Mtb-RNase J/ssRNA crystals were grown by mixing 1 µl of the above sample with 1 µl of reservoir solution, comprised of 7 % MPD and 0.1 M Bicine, pH 8.5. The RNase J crystals were obtained by mixing 1 µl of protein (17 mg/mL) with 1 µl of reservoir solution, comprised of a reservoir containing 4.3 M sodium chloride, 0.1 M HEPES pH7.5. Tiny crystals appeared within two days, and large crystals were obtained after several rounds of optimizations of pH and NaCl concentration. Finally, all the crystals were briefly soaked in a cryoprotectant solution consisting of 25-35% (vol/vol) glycerol dissolved in their corresponding reservoir before being flash-cooled directly in a liquid-nitrogen stream at 100 K. The X-ray diffraction data were collected at the BL17U1 and BL19U1 beamlines of the Shanghai Synchrotron Radiation Facility. Integration, scaling, and merging of the diffraction data were performed using the XDS program package and HKL2000.

### Structure determination

The crystal structure of Mtb-RNase J was determined by molecular replacement using *Deinococcus radiodurans* RNase J (PDB: 4XWW) structure as a search model. The RNase J/ssRNA complex structure was solved by using the Mtb-RNase J structure as a search model. Cycles of refinement and model building were carried out by using Phenix and COOT programs. The quality of the final model was evaluated with PROCHECK. All of the structures were displayed and analyzed using the PyMOL program. The collected data and refinement statistics are summarized in Table 1.

**Table 1:** Data collection and refinement statistics

	RNase J	RNase J-ssRNA
PDB code	7WNT	7WNU
*Data collection*		
Space group	P6422	C222_1_
Unit cell a,b,c (Å)	128.52 128.52 184.276	92.06 145.38 192.1
α, β, γ [°]	90 90 120	90 90 90
Resolution range (Å)^a^	44.4–2.45 (2.53–2.45)	41.51–3.20 (3.31–3.20)
Total reflections	67687 (6538)	43318 (4314)
Unique reflections	33845 (3269)	21660 (2157)
Completeness (%)	99.65 (98.58)	99.80 (99.72)
I/σ(I)	22.93 (2.43)	16.77 (5.24)
Wilson B-factor	58.61	59.25
R-merge^b^	0.016 (0.286)	0.040 (0.156)
R-meas	0.022 (0.404)	0.057 (0.221)
R-pim	0.016 (0.286)	0.040 (0.156)
CC1/2	1 (0.872)	0.998 (0.973)
*Refinement statistics*		
Resolution (Å)	44.4–2.45 (2.53–2.45)	41.51–3.20 (3.31–3.20)
Reflections used in refinement	33789 (3267)	21630 (2151)
Reflections used for R-free	1628 (139)	1067 (93)
*R*_work_^c^	0.219 (0.327)	0.185 (0.255)
*R*_free_^d^	0.251 (0.369)	0.240 (0.356)
No. residues	529	1111
*RMSD*		
Bond lengths (Å)	0.009	0.011
Bond angles (Å)	1.33	1.34
Average B-factor	63.79	63.03
Ramachandran favored (%)	96.18	89.57
Ramachandran allowed (%)	3.82	10.43
Ramachandran outliers (%)	0	0
*No. atoms*		
Protein or RNA	3882	8536
Ligands or ions	2	4
water	86	0
*B-factors*		
Protein or RNA	63.99	63.03
Ligands or ions	60.73	66.61

^a^Values in parentheses are for the highest resolution shell.

^b^*R*_merge_ = ∑|*I*_i_ −  > | /∑ | *I* | , where *I*_i_ is the intensity of an individual reflection and is the average intensity of that reflection.

^c^*R*_work_ = ∑||*F*_o_ | − |*F*_c_| |/∑|*F*_o_|, where *F*_o_ and *F*_c_ are the observed and calculated structure factors for reflections, respectively.

^d^*R*_free_ was calculated as *R*_work_ using the 5% of reflections that were selected randomly and omitted from refinement.

### Small angels X-ray scattering (SAXS)

The SAXS data of Mtb-RNase J were collected at beamline BL19U2 of the Shanghai Synchrotron Radiation Facility, with a radiation wavelength of 1.03 Å. The protein samples were prepared at around 2 mg/mL in a buffer containing 20 mM Tris-HCl (pH 8.0) and 100 mM NaCl. The samples were measured in triplicate, and the sample measurements were adjusted by subtracting the scattering from the buffer alone. Data were analyzed using the software package BioATSAS (https://www.embl-hamburg.de/biosaxs/). The scattering images were averaged and subtracted from the buffer-scattering images. Then, using the indirect Fourier transform method, the Rg was estimated. The distribution function p(r) was calculated from the parameter as Dmax. The SAXS envelope of Mtb RNase J was built by GASBOR, as previously reported^39^.

### Structure-based sequence alignment

Multiple alignments of amino acid sequences were performed using ClustalX v.2 program. Secondary structure alignment was generated by DSSP v.2.0 and ESpript v.3.0 (http://espript.ibcp.fr/ESPript/ESPript/).

### RNase J knockout and complementation in *Mycobacterium smegmatis* (Msm)

Msm-RNase J knockout plasmid was obtained by the homologous recombinant method. The upstream (2,760,206–2,762,205) and downstream (2,763,883–2,765,882) homologous fragments flanking the RNase J were cloned from the genome of Msm MC^2^155, then constructed into the plasmid containing the resistant gene and SacB gene by ClonExpress® MultiS kit (Vazyme, China). The correct clones were confirmed by restriction enzyme digestion and DNA sequencing. Electroporation of Msm MC^2^155 was performed as previously described^40^. After electroporation and recovery, cells were plated on 7H10 plates containing 50 µg/mL kanamycin to screen the positive clones. Then, the positive monoclones were transferred to a non-resistance 7H19 medium for double exchange and plated on 7H10 plates containing 50 µg/mL kanamycin and 10% sucrose for another three days. The correct RNase J knockout (KO) strain was confirmed by the colony PCR method. The RNase J complemented strain was obtained by transforming the pMV261 plasmid expressing RNase J into the KO strain and obtained the RNase J stable-expressing Msm strain through single-clone screening and identification. The correct RNase J complemented (Com) strain was confirmed by colony PCR and qRT-PCR methods.

### Growth curve

The wild-type (WT), RNase J knockout, and RNase J complemented strains were cultured to OD_600_ of 0.6, and then added to 50 mL 7H9 medium at a ratio of 1:200 for culture. OD_600_ was measured every three hours. Three repetitions were performed for each group.

### Plate colony growth

The *Mycobacterium smegmatis* strains were cultured to OD_600_ of 0.6, then diluted to 10^−5^, 10^−6^, and 10^−7^ times, respectively. Then the cells were incubated on non-resistance 7H10 plates at 37 °C. The colony size and morphology were recorded every day. In addition, the colony morphology was observed by stereoscopic microscope.

### Transmission electron microscope

The *Mycobacterium smegmatis* strains were cultured to OD_600_ of 0.6 and washed with double-distilled water three times, which were then fixed with 2.5% glutaraldehyde and 4% paraformaldehyde at room temperature for around 15 min. The fixed bacteria were covered on a 200-mesh carbon film, stained with uranyl acetate. The morphologies of the bacteria were observed and photographed with a 120 kV transmission electron microscope.

### Total RNA sequencing and analyzing

The WT and KO strains of *Msm*-RNase J were cultured to OD_600_ of 0.8. The total RNA of each sample was extracted using the TRIzol Reagent (Invitrogen). The total RNA with an RNA integrity number (RIN) value above nine was used for library preparation. The library for next-generation sequencing was constructed according to the manufacturer’s protocol (NEBNext®Ultra™RNA Library Prep Kit for Illumina®). The libraries with different indices were multiplexed and loaded on an Illumina novaseq6000 instrument according to the manufacturer’s instructions (Illumina, San Diego, CA, USA). Sequencing was carried out using a 2 × 150 -bp paired-end (PE) configuration. The raw paired-end reads obtained from RNA-seq experiments were filtered out low-quality reads and then aligned to the Msm MC^2^155 genome sequence using the STAR with default settings. To choose genes with accurate expression values, we consider genes whose FPKM > 1 in at least one sample for subsequent analysis. Differential gene analysis was performed using edgeR. The statistical cutoff of FDR < 0.05 and fold change>2 was applied to obtain differentially regulated genes. The top five enrichment pathways with adjusted *P*-values <0.05 were selected.

### RNA extraction and determination of mRNA abundance and stability

The wild-type (WT), RNase J knockout (KO), and RNase J complemented (COM) strains were cultured to OD600 of 0.6 and harvested at 4000 rpm for 10 min. RNA was purified using the RNAprep Pure Cell/Bacteria Kit (TIANGEN) according to the manufacturer’s instructions. The cDNA was synthesized using the HiScript III 1st Strand cDNA Synthesis Kit (Vazyme) according to the manufacturer’s instructions. RNA abundance was determined by quantitative PCR (qPCR) using the ChamQ Universal SYBR qPCR Master Mix (Vazyme) on the Applied Biosystems® QuantStudio™ 7 Flex Real-Time PCR System (ThermoFisher Scientific) according to the manufacturer’s instruction. Primers used in this study are listed in Table S4. For mRNA stability analysis, WT or KO strain cultures were treated with rifampin at a final concentration of 150 μg/mL to halt transcription and snap-frozen in liquid nitrogen after 0, 1, 2, 5, 10, or 20 min. Abundance over time was determined using qPCR.

### Statistical analysis

Each experiment was performed at least three times. All experiment data were analyzed using GraphPad Prism 7.0 (GraphPad software Inc. USA) and were presented as mean values ± SD. Statistical analysis was performed using a *t* test (*p < 0.05; **p < 0.01; ***p < 0.001).

### Reporting summary

Further information on research design is available in the Nature Portfolio Reporting Summary linked to this article.

## Supplementary information

Supplementary Information

Reporting Summary

## Data Availability

The data supporting the findings of this study are available from the corresponding authors upon reasonable request. The coordinates and structural factors generated in this study have been deposited in the Protein Data Bank with accession codes 7WNT (Mtb-RNase J-apo) and 7WNU (Mtb-RNase J-ssRNA complex). The bulk RNA-seq data generated in this study has been deposited in the Sequence Read Archive (SRA) database with accession number PRJNA792207. Source data are provided with this paper.

## Source data

Source Data
