# Supplementary material for: Structural insights into RNase J that plays an essential role in Mycobacterium tuberculosis RNA metabolism
Source: Nat Commun. 2023 Apr 20;14:2280. doi: 10.1038/s41467-023-38045-z (PMC10119312; doi:10.1038/s41467-023-38045-z)
Supplement: Supplementary file 1 — Supplementary Information [file 41467_2023_38045_MOESM1_ESM.pdf]

Supplementary Information For

Structural insights into RNase J that plays an essential role in Mycobacterium tuberculosis RNA metabolism

Luyao Bao[1,#], Juan Hu[1,#], Bowen Zhan[1,#], Mingzhe Chi[1], Zhengyang Li[1], Sen Wang[1], Chan Shan[2], Zhaozhao Zhao[2], Yanchao Guo[1], Xiaoming Ding[2], Chaoneng Ji[1], Shengce Tao[3], Ting Ni[2], Xuelian Zhang[2,*], Guoping Zhao[2,4,*], Jixi Li[1,5,*]

[*]To whom correspondence should be addressed. Email: lijixi@fudan.edu.cn or xuelianzhang@fudan.edu.cn or gpzhao@sibs.ac.cn

This PDF file includes:

Supplementary Figure 1 to 9

Supplementary Tables 1 to 4

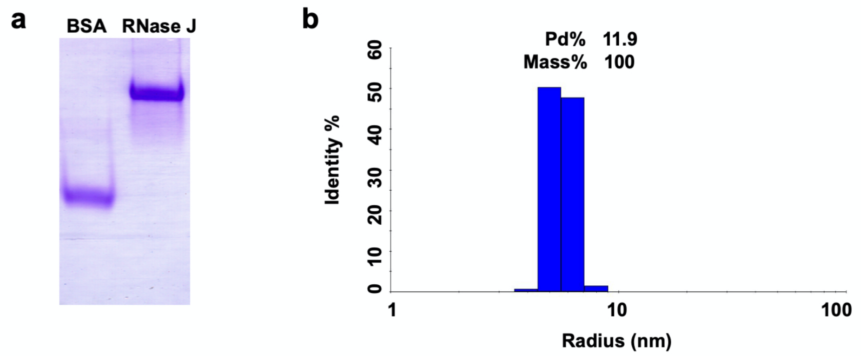

c

Protein	*Km* (μM)	*Kcat* (min^{-1})
RNase J	111.10± 16	0.023± 0.012
BlaC	57	6670

d

Ions	*Km* (μM)	*Kcat* (min^{-1})	*Kcat /Km* ($min^{-1}μM^{-1}$)
No	1.78±0.10	1.44±0.025	0.82
Mn^{2+}	0.32±0.072	3.86±0.31	12.06

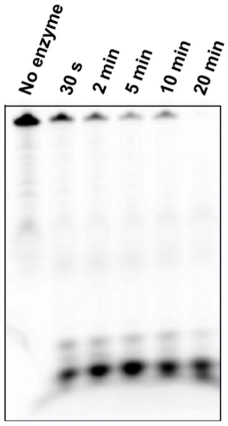

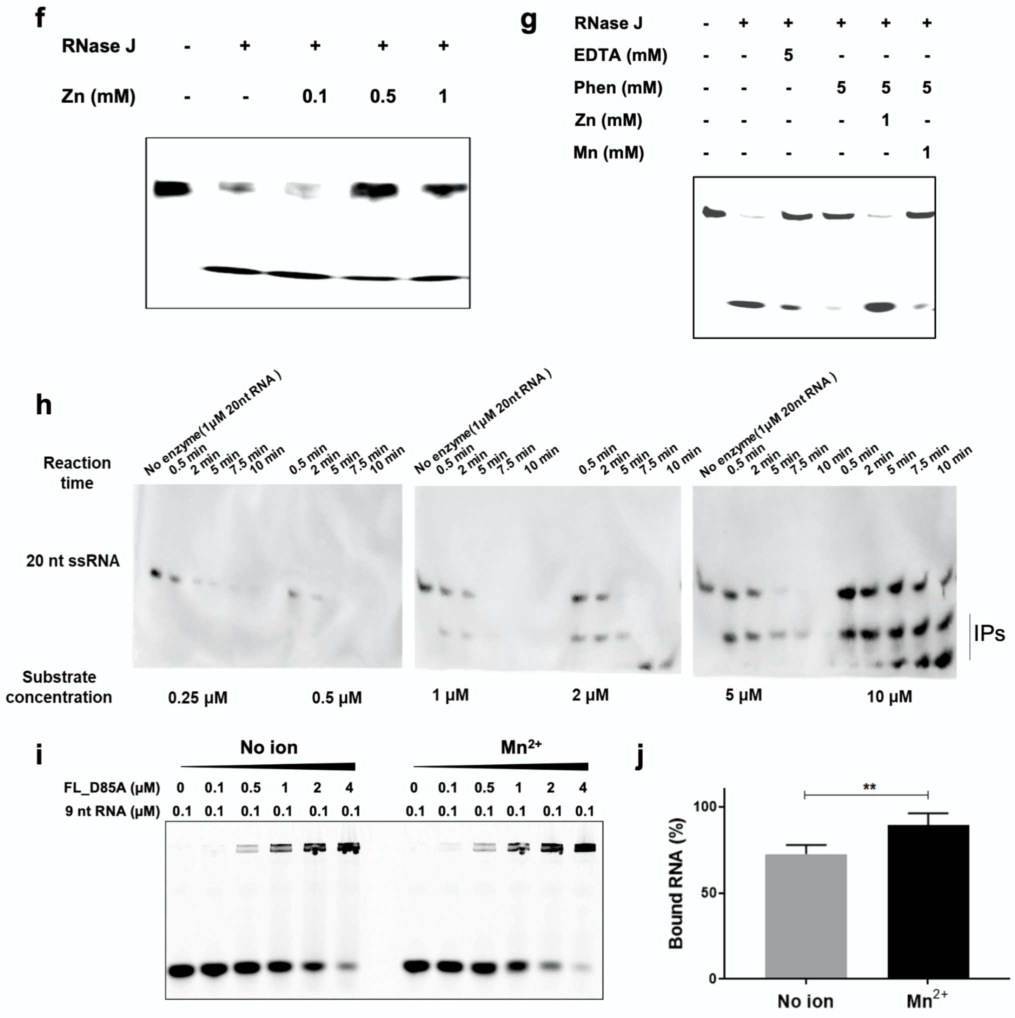

Supplementary Figure 1. Biochemical properties of Mtb-RNase J, related to Figure 1. a. Native-PAGE analysis of Mtb-RNase J. **b.** Dynamic light scattering (DLS) analysis of Mtb-RNase J. **c.** β-lactamase activities comparison between Mtb-RNase J and Mtb-BlaC. **d.** Ribonuclease activities comparison between Mtb-RNase J alone and with the presence of Mn^{2+}. **e.** The endoribonuclease activities of Mtb-RNase J were assayed with a 3'-FAM labeled 20-nt poly(U) as the substrate. **f**. The ribonuclease activity of Mtb-RNase J with varying concentrations of Zn^{2+} (0.1 mM, 0.5 mM, and 1 mM). **g.** Mtb-RNase J was chelated with EDTA or Phen, then supplemented with Mn^{2+} or Zn^{2+}. **h.** The ribonuclease activity of Mtb-RNase J with varying concentrations (0.25 μM, 0. 5 μM, 1 μM, 2 μM, 5 μM, and 10 μM) of 20-nt poly(U) RNA as substrate. IPs: intermediate products. **i.** EMSA assay of Mtb-RNase J with a 9-nt poly(A) RNA substrate either with or without Mn^{2+}. The concentration of Mn^{2+} and RNA are 5 mM and 0.1 μM, respectively. The mutant enzyme D85A was used to do the EMSA assay. **j.** Quantification of bound-RNA intensity in **f** with three times repetition.

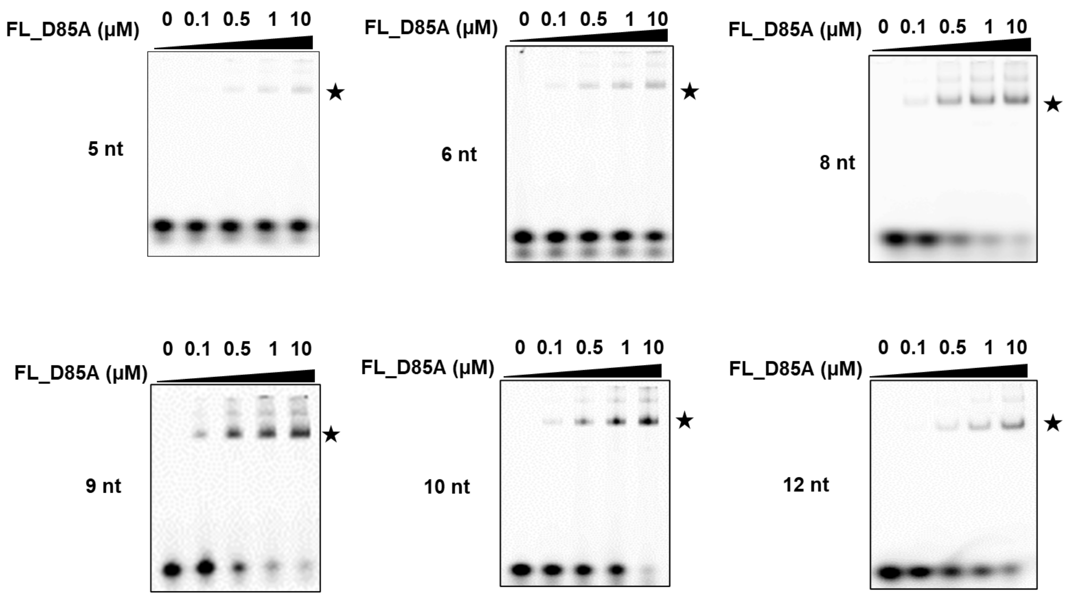

Supplementary Figure 2. Full-length Mtb-RNase J prefers to bind with 9-nt single-strand RNA. The asterisks showed the bound RNA. RNA concentration is 0.5 μM.

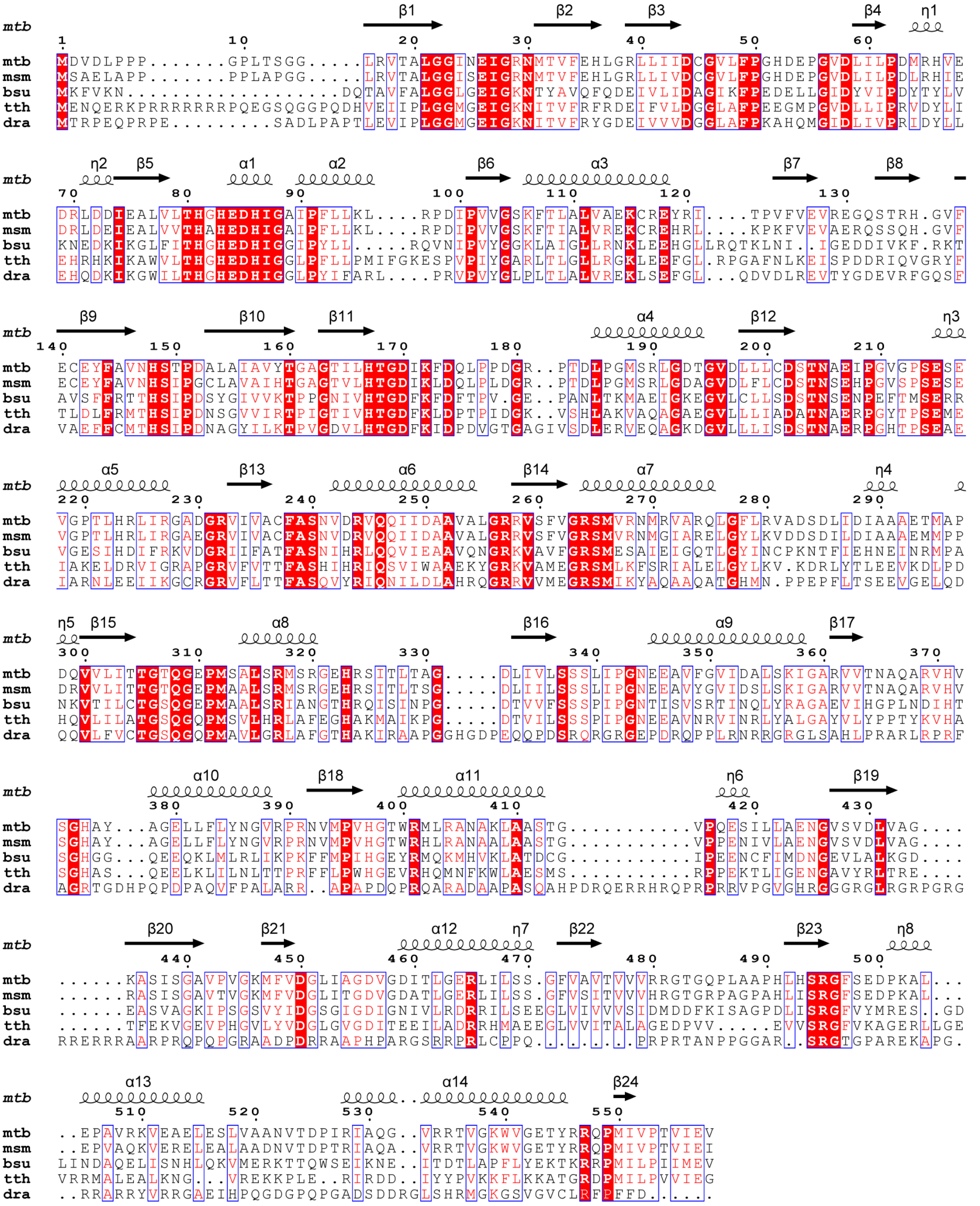

Supplementary Figure 3. Structure-based sequence alignment of Mtb-RNase J with other homologous proteins. Sequence alignment was performed using the ClustalX and ESPript programs. Names of different species are shown as mtb, *Mycobacterium tuberculosis*; msm, *Mycobacterium smegmatis*; bsu, *Bacillus subtilis*; tth, *Thermus thermophilus*; dra, *Deinococcus radiodurans*. Identical and similar residues among groups are shown in white text on a red

background and red text on a white background, respectively. The second structure elements, including α-helix, β-strand, and 3_{10}-helix, are denoted as α, β, and η, respectively.

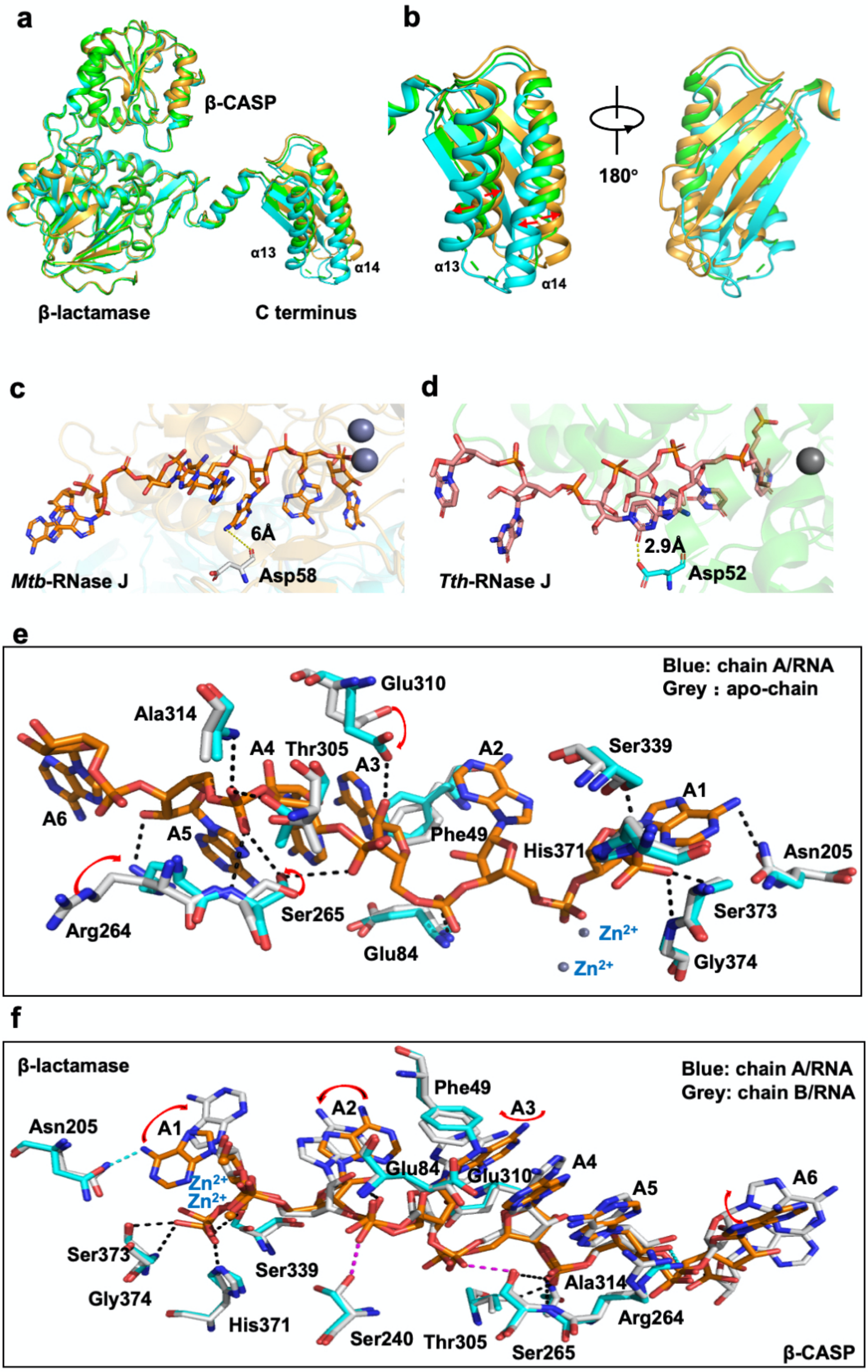

Supplementary Figure 4. Conformational change upon RNA binding, related to Figure 3. a-b. Conformational change upon RNA binding of Mtb-RNase J. The apo structure was shown in

green. Chains A and B in the Mtb-RNase J/ssRNA complex were shown in orange and cyan, respectively. **c-d.** Structural superimposition of Mtb-RNase J and Tth-RNase J. **e.** Residues involved in RNA-binding and conformational changes were shown in stick models. The chain A in Mtb-RNase J/ssRNA and the apo structure Mtb-RNase J were colored blue and grey, respectively. **f.** The formational difference between chain A and chain B. Residues involved in RNA binding was shown in stick models. The chains A and B in the Mtb-RNase J/ssRNA complex were colored blue and grey, respectively.

a

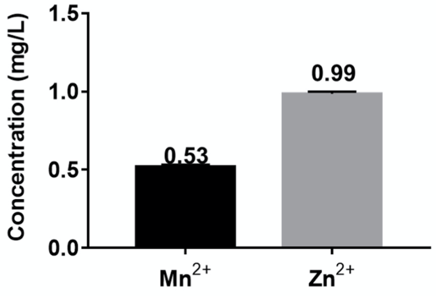

b

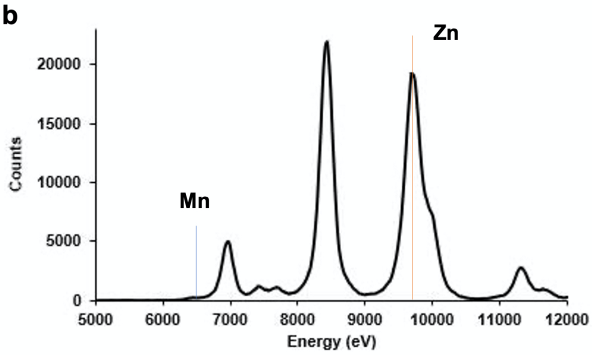

Supplementary Figure 5. Zinc ion was identified in the RNase J active site. a. ICP-MS analysis of the fresh-purified Mtb-RNase J protein (~1 mg/mL). **b.** X-ray fluorescence scanning of Mtb-RNase J crystals at the synchrotron gave a strong peak at the ZnK-Edge (9600 eV).

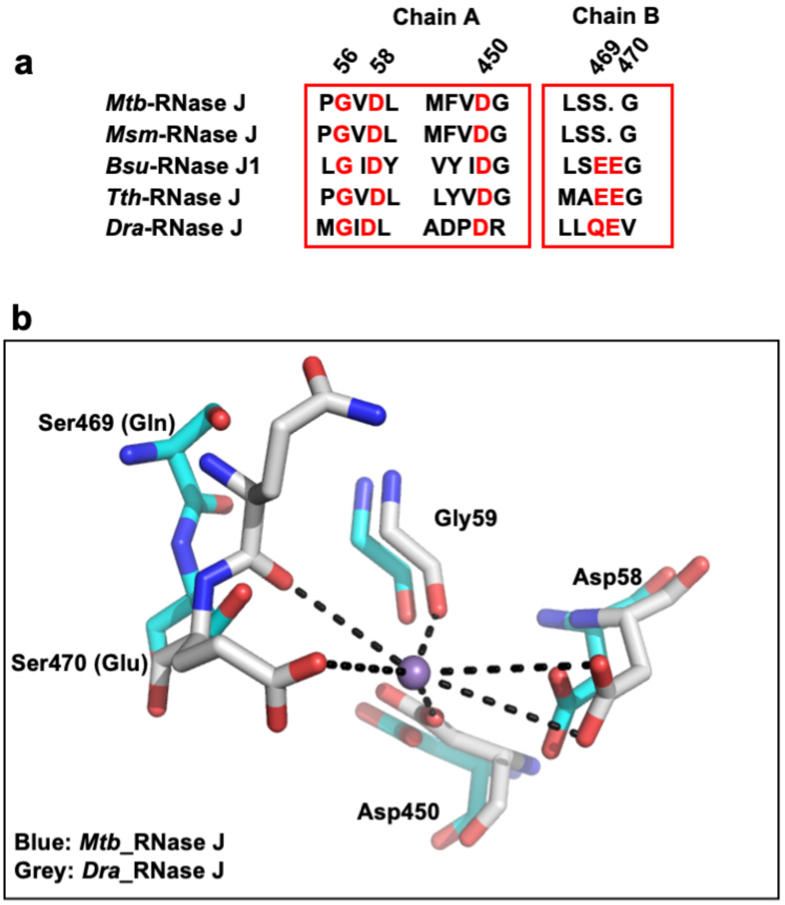

Supplementary Figure 6. The role of divalent manganese in Mtb-RNase J dimerization. a. Homologous sequence alignment of the dimeric interface-related residues in different species. **b.** Structural differences between Mtb-RNase J and Dra-RNase J at the dimeric interface. Residues were shown in stick models. Mn^{2+} was shown in a purple sphere.

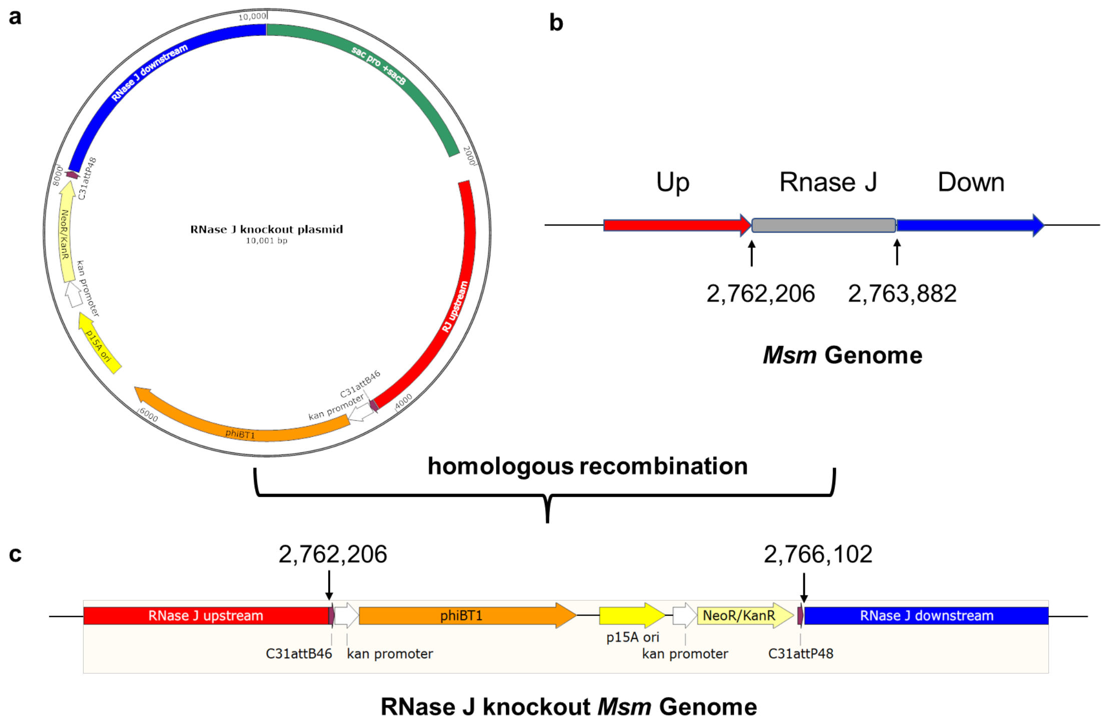

Supplementary Figure 7. Schematic diagram of RNase J knockout by homologous recombination. a. Map of Msm-RNase J gene knockout plasmid. **b.** The location of RNase J gene on *Mycobacterium smegmatis* genome. **c.** Schematic of *Mycobacterium smegmatis* genome after RNase J knockout.

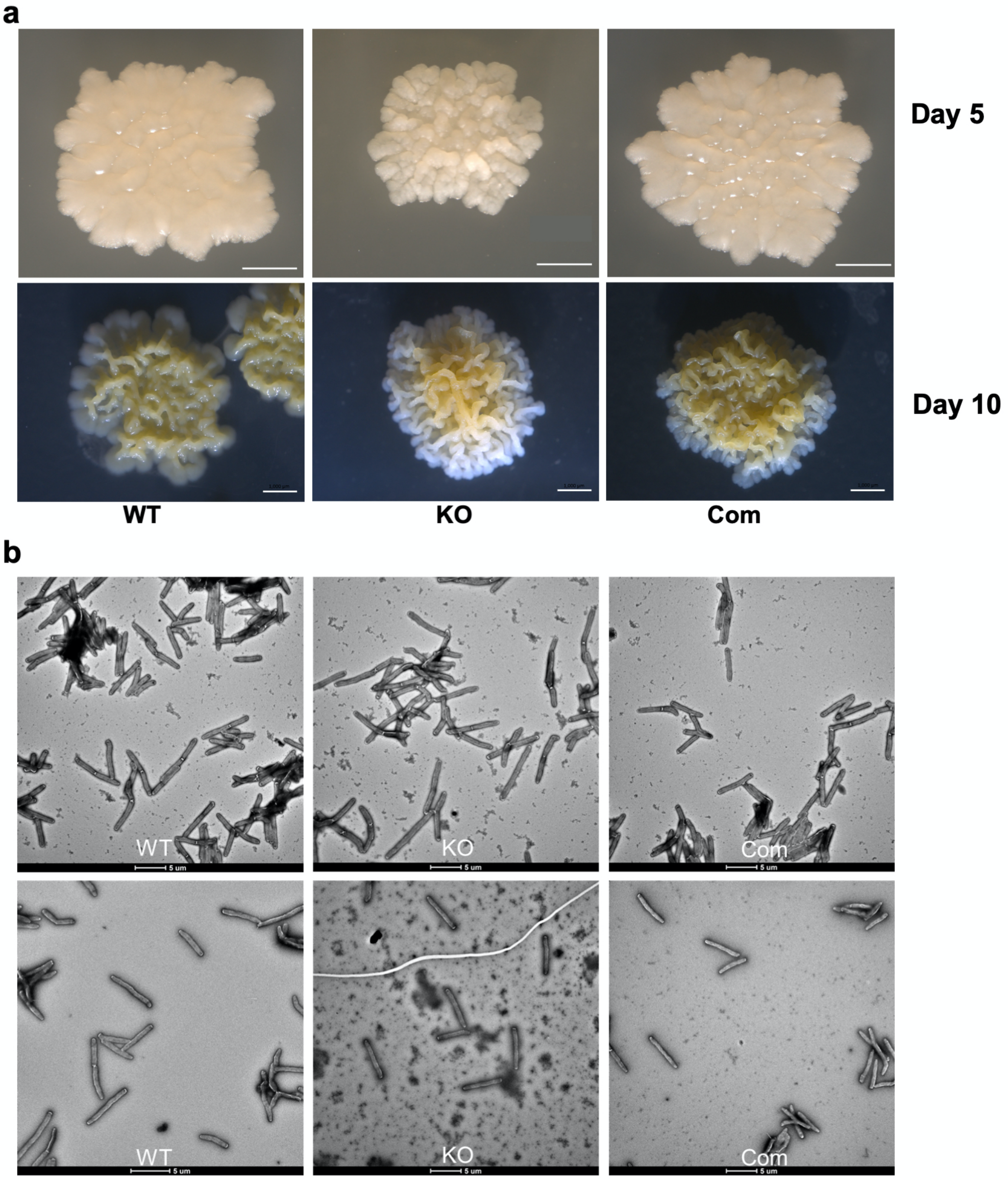

Supplementary Figure 8. RNase J affects the growth and morphology of *Mycobacterium smegmatis* by affecting cell wall synthesis and lipid metabolism, related to Figure 7. a. Colony morphologies of wild-type (WT), knockout (KO), and RNase J complemented (Com) strains on 7H10 solid medium at day 5 and day 10. Scale bar, 1 mm. **b.** Cell length of WT, KO, and RNase J Com strains. Scale bar, 5 µm.

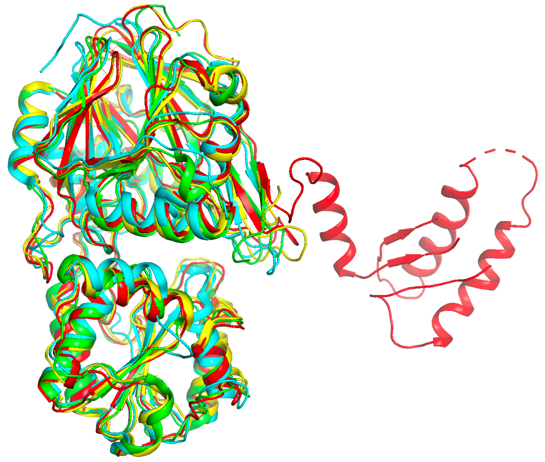

Supplementary Figure 9. Structural superimposition of Mtb-RNase J (PDB ID: 7WNT, red), *S. coelicolor* RNase J (PDB ID: 5A0T, yellow), *S. epidermidis* RNase J1 (PDB ID: 6K6S, green), and RNase J2 (PDB ID: 6K6W, cyan).

Supplementary Table 1. The putative RNases are presented in different species.

	E.coli	*B.subtilis*	*M.smegmatis*	*M.tuberculosis*
**Endoribonucleases**				
RNase E	+	-	+	+
RNase G	+	-	-	-
RNase III	+	+	+	+
Mini III	-	+	-	-
RNase M5	-	+	-	-
RNase P	+	+	+	+
RNase Z	+	+	+	+
RNase I	+	-	-	-
RNase Y	-	+	-	-
RNase J	-	+	+	+
**Exoribonucleases**				
RNase J	-	+	+	+
PNPase	+	+	+	+
RNase T	+	-	-	-
RNase R	+	+	-	-
RNase II	+	-	-	-
RNase PH	-	+	+	+
YhaM	-	+	-	-
Orn	+	-	+	+
NrnA	-	+	+	+

Supplementary Table 2. Molecular interactions in the C-terminus of Mtb-RNase J.

Polar interactions in the C-terminus of Mtb-RNase J				
Bonds	No	Chain A	Distance（Å）	Chain B
Hydrogen bonds	1	A:LEU 452[N]	2.94	B:TYR 545[O]
	2	A:ILE 460 [N]	3.27	B:GLU 499[OE2]
	3	A:THR 461[N]	3.19	B:GLU 499[OE2]
	4	A:THR 461[OG1]	2.86	B:GLU 499[OE2]
	5	A:GLU 464[OE2]	2.36	B:GLY 496[N]
	6	A:ASP 456[O]	3.09	B:GLU 499[N]
	7	A:TYR 545[O]	2.94	B:LEU 452[N]
	8	A:GLU 499[OE2]	3.27	B:ILE 460[N]
	9	A:GLU 499[OE2]	3.19	B:THR 461[N]
	10	A:GLU 499[OE2]	2.86	B:THR 461[OG1]
	11	A:GLY 496[N]	2.36	B:GLU 464[OE2]
	12	A:GLU 499[N]	3.09	B:ASP 456[O]
Salt bridges	1	A:ARG 547		B:ASP 450
	2	A:ARG 547		B:ASP 456
	3	A:ASP 450		B:ARG 547
	4	A:ASP 456		B:ARG 547

Supplementary Table 3. Molecular interactions in the dimeric interface between the β-CASP and the β-lactamase domain.

Polar contacts in the dimer interface between β-CASP and β-lactamase				
Bonds	No	Chain A	Distance(Å)	Chain B
Hydrogen bonds	1	A:TRP 400[N]	3.44	B:ASP 353[OD1]
	2	A:ARG 401[N]	3.15	B:ASP 353[OD1]
	3	A:ARG 404[NE]	2.64	B:SER 356[O]
	4	A:ARG 404[NH2]	2.58	B:LYS 357[O]
	5	A:ASP 353[OD1]	3.44	B:TRP 400[N]
	6	A:ASP 353[OD1]	3.15	B:ARG 401[N]
	7	A:SER 356[O]	2.64	B:ARG 404[NE]
	8	A:LYS 357[O]	2.58	B:ARG 404[NH2]
Salt bridges	1	A:LYS 357		B:GLU 424
	2	A:ARG 401		B:ASP 353
	3	A:GLU 424		B:LYS 357
	4	A:ASP 353		B:ARG 401

Supplementary Table 4. Q-PCR primers were listed below.

Gene Name	F-primer	R-primer
RNase J	TGTTGATCACCACGGGAACC	TTGGGCATTGGTGACCACTC
SigA	CGTCCGGCGACTTCGTGT	TGGCCAGCTCCACCTCTTCT
MSMEG_2686	CACCTCCAACGCGACATACG	ACACGGTCGAGGTGGTCTTC
MSMEG_2687	CACCGCTTGTTGACGATGCC	CGATGATCGACAACGAGTTC
MSMEG_0550	GCGATCGGAACGATCTTCTC	GGCGGTGGTGAACAGCTATG
MSMEG_5790	GTTGACCTGGAGAAGGAAAC	GCGGCGAAGAACCGGAAATC
MSMEG_3652	AGCGGGAATCCCGACATCAG	CGCTGGTGGATCGCATCTAC
MSMEG_4157	GACGAGATCAACCGCATCCG	AGGGCATGAGCGAGTTGGTG
MSMEG_0275	TCGGTTCCTGAGAGGTACAC	TCGACCACCAGCACGCATTC
MSMEG_2856	ACCGCACTGGACAACGGAAC	GCACCAGTTTGCGGTAGTTG
MSMEG_2166	TTCGCCGAGTCGGTATCCAG	TCCTGAGGCACTTCGACAAC
MSMEG_6855	GTATTTCTCGCCGAGGGCAG	GCTGCTCAACCTCGTCATCG
MSMEG_2888	GCGGCGATTCCGATGGATTC	TGATGAGGGCCTGGCCTATG
